# Blockade of checkpoint receptor PVRIG unleashes anti-tumor immunity of NK cells in murine and human solid tumors

**DOI:** 10.1186/s13045-021-01112-3

**Published:** 2021-06-26

**Authors:** Yangyang Li, Yu Zhang, Guoshuai Cao, Xiaodong Zheng, Cheng Sun, Haiming Wei, Zhigang Tian, Weihua Xiao, Rui Sun, Haoyu Sun

**Affiliations:** 1 Hefei National Laboratory for Physical Sciences at Microscale, the CAS Key Laboratory of Innate Immunity and Chronic Disease, School of Basic Medical Sciences, Division of Life Sciences and Medicine, University of Science and Technology of China, 443 Huangshan Road, Hefei, 230027 China; 2 Institute of Immunology, University of Science and Technology of China, Hefei, China; 3 Research Unit of NK Cell Study, Chinese Academy of Medical Sciences, Beijing, China; 4 Hefei TG ImmunoPharma Corporation Limited, Hefei, China

**Keywords:** PVRIG, CD112R, NK cell, Immune checkpoint blockade, Anti-tumor immunotherapy

## Abstract

**Background:**

Although checkpoint-based immunotherapy has shown exciting results in the treatment of tumors, around 70% of patients have experienced unresponsiveness. PVRIG is a recently identified immune checkpoint receptor and blockade of which could reverse T cell exhaustion to treat murine tumor; however, its therapeutic potential via NK cells in mice and human remains seldom reported.

**Methods:**

In this study, we used patient paraffin-embedded colon adenocarcinoma sections, various murine tumor models (MC38 colon cancer, MCA205 fibrosarcoma and LLC lung cancer), and human NK cell- or PBMC-reconstituted xenograft models (SW620 colon cancer) to investigate the effect of PVRIG on tumor progression.

**Results:**

We found that PVRIG was highly expressed on tumor-infiltrating NK cells with exhausted phenotype. Furthermore, either PVRIG deficiency, early blockade or late blockade of PVRIG slowed tumor growth and prolonged survival of tumor-bearing mice by inhibiting exhaustion of NK cells as well as CD8^+^ T cells. Combined blockade of PVRIG and PD-L1 showed better effect in controlling tumor growth than using either one alone. Depletion of NK or/and CD8^+^ T cells in vivo showed that both cell types contributed to the anti-tumor efficacy of PVRIG blockade. By using *Rag1*^*−/−*^ mice, we demonstrated that PVRIG blockade could provide therapeutic effect in the absence of adaptive immunity. Further, blockade of human PVRIG with monoclonal antibody enhanced human NK cell function and inhibited human tumor growth in NK cell- or PBMC-reconstituted xenograft mice.

**Conclusions:**

Our results reveal the importance of NK cells and provide novel knowledge for clinical application of PVRIG-targeted drugs in future.

**Supplementary Information:**

The online version contains supplementary material available at 10.1186/s13045-021-01112-3.

## Background

Traditional therapies to treat cancer include chemotherapies and radiotherapies; although they are effective against some tumors, they also bring unpleasant side effects due to their unspecific attacks on normal cells and tissues. In recent years, immunotherapies involving checkpoint blockade have achieved huge success, checkpoint blockade is target-specific with much less side effects, and they unleash the power of immune cells [1, 2]. A variety of checkpoint inhibitors have been approved for the treatment of tumors [3–10]. Although these treatments are effective in some patients, there is still a large percentage of patients who do not respond well to these treatments and cannot benefit from these existing checkpoint immunotherapies.

A family of inhibitory receptors that interact with nectin and nectin-like family molecules has gained increasing attention [11–13], including TIGIT, CD96, PVRIG and the costimulatory receptor CD226, which share the same ligands CD155 and PVRL2 [14]. The interaction between TIGIT and CD155/PVRL2 suppresses anti-tumor and anti-viral immune responses in both direct and indirect manners [15]. High expression of TIGIT leads to the exhaustion of CD8^+^ T cells and NK cells [16, 17], and its expression is associated with the prognosis of tumor patients [18–20]. On the other hand, CD96 plays an important role in mouse lung metastasis and subcutaneous tumor models [21, 22]. Human CD96^+^ NK cells are functionally exhausted with impaired IFN-γ and TNF-α production [23]. PVRIG, also known as CD112R, has been discovered in the year of 2016 [24]. Being a recently discovered inhibitory receptor in this family, researches on this receptor are very limited compared with those on TIGIT, CD96 and CD226. PVRIG expresses on T cells and NK cells, and its expression on T cells increases with cell activation [24]. Upregulation of PVRIG on CD8^+^ T cells causes their exhaustion in lymphocytic choriomeningitis virus infection [25]. CD8^+^ T cells from PVRIG-deficient mice show stronger antigen-specific effector functions during acute *Listeria monocytogenes* infection [26]. Furthermore, PVRIG-deficient mice display significantly reduced tumor growth due to enhanced CD8^+^ T cell function [26].

Besides CD8^+^ T cells, NK cells are also essential anti-tumor effector cells [27]. The introduction of the term “cold tumor” leads to the boost emergence of anti-tumor immunotherapies involving NK cells, which are particularly important for treating cytotoxic T lymphocyte (CTL)-insensitive tumors with no or minimum MHC class I expression. Reduced NK cell number or impaired NK cell function has been associated with the progression of various types of cancers [28, 29]. It has been reported that blocking PVRIG not only promotes cytokine secretion and proliferation of human T cells, but also enhances antibody-dependent cell-mediated cytotoxicity (ADCC) of human NK cells [24, 30]. In addition, PVRIG blockade enhances NK cell killing of its ligand PVRL2^hi^ acute myeloid cells [31]. However, the role of PVRIG in the regulation and immunotherapy of NK cells in the solid tumor microenvironment has not been investigated.

In this study, we generated a rat anti-mouse PVRIG monoclonal antibody (mAb) that specifically blocks the interaction between PVRIG and its ligand PVRL2. Genetic knock-out of PVRIG in mice or treatment with anti-PVRIG mAb (both early and late treatments) significantly inhibited the exhaustion of NK cells and slowed tumor growth in several murine tumor models. We showed that besides CD8^+^ T cells, the presence of NK cells was also critical for the therapeutic effects of PVRIG blockade. Furthermore, we generated mouse anti-human PVRIG mAb and found that anti-human PVRIG (anti-hPVRIG) slowed tumor growth in both human NK cell- and peripheral blood mononuclear cell (PBMC)-reconstituted xenograft murine models. These findings indicate that blockade of PVRIG not only promotes the anti-tumor immunity of CD8^+^ T cells, but also unleashes the anti-tumor power of NK cells, therefore making PVRIG a promising immune checkpoint target to treat cancer.

## Methods

### Mice

C57BL/6J mice were purchased from Shanghai Experimental Animal Center (Shanghai, China) or GemPharmatech Corporation Limited (Nanjing, China). *Rag1*^*−/−*^ mice were purchased from GemPharmatech Corporation Limited (Nanjing, China). C57BL/6 *Pvrig*^*+/–*^ mice were generated by Beijing Biocytogen Corporation Limited (Beijing, China), and *Pvrig*^*−/−*^ mice were bred in-house. B-NDG mice (NOD.CB17-*Prkdc*^*scid*^*Il2rg*^*tm1*^/Bcgen) were purchased from Beijing Biocytogen Corporation Limited (Beijing, China). All mice were maintained in a specific pathogen-free facility for use. Mice were used between 6 and 8 weeks of age. Animal experiments were approved by the ethics committee of the University of Science and Technology of China.

### Cell lines

Lewis Lung Carcinoma (LLC) cell line was purchased from the cell bank of the Chinese Academy of Science (Shanghai, China). MCA205 fibrosarcoma cell line was purchased from BNCC (Beijing, China). MC38 colon adenocarcinoma cell line was kindly provided by Professor Yangxin Fu from University of Texas Southwestern Medical Center (Dallas, USA). Hybridoma cells PK136 (anti-NK1.1; in vivo depletion) were purchased from ATCC (Manassas, USA). Clone 1 (rat IgG1,κ), a blocking monoclonal antibody to mouse PVRIG, was a novel clone generated in-house by our laboratory with no prominent ADCC. Clone 2 (mouse IgG1,κ), a blocking monoclonal antibody to human PVRIG, was a novel clone generated in-house by our laboratory with no prominent ADCC. All cell lines were maintained in DMEM medium containing 10% FBS. SW620 human colon cancer cell line, A375 human melanoma cell line and SK-OV-3 human ovarian cancer cell line were purchased from the cell bank of the Chinese Academy of Science (Shanghai, China). SW620 cell line was maintained in L-15 medium containing 10% FBS. A375 cell line was maintained in DMEM medium containing 10% FBS. SK-OV-3 cell line was maintained in McCoy's 5a medium containing 10% FBS. NKG cell lines were established and maintained as previously described [32]. All cell lines were tested negative for mycoplasma contamination.

### Identification of anti-PVRIG antibodies

For rat anti-mouse PVRIG monoclonal antibody (Clone 1) binding assay, 2 × 10^5^ 293T-mouse PVRIG cells were incubated with different concentrations of antibodies (Clone 1), and then the binding frequency was detected using APC-conjugated goat anti-rat IgG antibody (Poly4054, Biolegend, San Diego, USA). For assessing PVRIG-PVRL2 antagonistic activity, 2 × 10^5^ 293T-mouse PVRIG cells were incubated with different concentrations of antibodies (Clone 1) and 10 μg/mL mouse PVRL2-hFc fusion protein. APC-conjugated mouse anti-human IgG Fc antibody (HP6017, Biolegend, San Diego, USA) was used to detect the binding frequency of mCD112-hFc fusion protein.

For mouse anti-human PVRIG monoclonal antibody (Clone 2) binding assay, 2 × 10^5^ 293T-human PVRIG cells were incubated with different concentrations of antibodies (Clone 2), and then the binding frequency was detected using APC-conjugated goat anti-mouse IgG antibody (Poly4053, Biolegend, San Diego, USA). For assessing PVRIG-PVRL2 antagonistic activity, 2 × 10^5^ 293T-human PVRL2 cells were incubated with different concentrations of antibodies (Clone 2) and 10 μg/mL human PVRIG-Fc fusion protein. APC-conjugated mouse anti-human IgG Fc antibody (HP6017, Biolegend, San Diego, USA) was used to detect the binding frequency of human PVRIG-Fc fusion protein.

### Surface plasmon resonance (SPR)

SPR measurements were performed on the Biacore 8 K high-throughput molecular interaction detection system (GE Healthcare, Little Chalfont, UK). The mouse PVRIG-Fc fusion protein or human PVRIG-Fc fusion protein was immobilized on a CM5 sensor chip (GE Healthcare, Little Chalfont, UK) under 25 degrees according to the manufacturer’s instructions. Anti-PVRIG antibody was flowed at increasing concentrations in the running buffer at 30 μL/min. The sensor chip was regenerated with 50 mM NaOH for every cycle. Specific binding of anti-PVRIG antibody to antigen was calculated automatically using the response to a blank channel as a reference. All the raw sensogram data were processed and fit using the Biacore 8 K Evaluation software version 1.1. (GE Healthcare, Little Chalfont, UK).

### Transplant tumor models

For early antibody treatment experiment, C57BL/6 mice or *Rag1*^*−/−*^ mice were inoculated subcutaneously with 5 × 10^4^ MC38 cells. Mice were randomized into treatment groups 3 days later and treated with anti-PVRIG (250 μg; purified in-house from Clone 1 hybridoma cell supernatant), isotype-matched control antibody (250 μg; purified in-house from rat serum) or PBS by intraperitoneal injection for six times (once every 3 days). For late antibody treatment experiment, C57BL/6 mice were inoculated subcutaneously with 2 × 10^5^ MC38 cells. Mice were randomized into treatment groups when tumor size reaches 100–150 mm^3^ and treated with anti-PVRIG (250 μg; purified in-house from Clone 1 hybridoma cell supernatant) or isotype-matched control antibody (250 μg; purified in-house from rat serum) by intraperitoneal injection for six times (once every 3 days). To evaluate the effect of combined therapy, C57BL/6 mice were treated intraperitoneally with isotype-matched control antibody (250 μg), anti-PD-L1 (100 μg; 10F.9G2, Bio X Cell, Lebanon, USA), anti-PVRIG (250 μg) or anti-PD-L1 (100 μg) combined with anti-PVRIG (250 μg) for six times (once every 3 days) starting on day 3. To evaluate the tumor growth in wild-type and *Pvrig*^*−/−*^ mice, mice were inoculated subcutaneously with 5 × 10^4^ MCA205 cells, 2 × 10^5^ MC38 cells or 1 × 10^6^ LLC cells.

For human NK cell-reconstituted xenograft model, female B-NDG mice were inoculated subcutaneously with 1 × 10^6^ SW620 colon cancer cells on day 0. Mice were grouped randomly and received 1 × 10^7^ expanded human NK cell transfer on days 7, 12 and 17, along with control antibody treatment or anti-human PVRIG mAb (250 μg; purified in-house from Clone 2 hybridoma cell supernatant) on days 7, 10, 13, 16 and 19. All mice were injected intraperitoneally with 50,000 IU recombinant human IL-2 every two days starting on day 7. For human PBMC-reconstituted xenograft model, female B-NDG mice were inoculated subcutaneously with 1 × 10^6^ SW620 colon cancer cells on day 0. Mice were grouped randomly and received 1 × 10^7^ human PBMC transfer on day 7. After that, mice were treated with PBS, control antibody or anti-human PVRIG mAb (250 μg) by intraperitoneal injection for five times (once every 3 days). Tumors were measured every two or three days by caliper, and tumor volume was calculated as 0.5 × length × width × width. Mice were euthanized when tumor size reaches 1000 mm^3^.

### Isolation of tumor-infiltrating lymphocytes (TILs)

TILs were isolated by dissociating tumor tissue in the presence of collagenase IV (1 mg/mL, Sigma-Aldrich, St. Louis, USA) and DNase I (15 U/mL, Sigma-Aldrich, St. Louis, USA) for 1 h before centrifugation on a discontinuous Percoll gradient (GE Healthcare, Little Chalfont, UK). Isolated cells were then used in various assays to evaluate the phenotype and function of NK cells and T cells.

### Antibodies

Monoclonal antibodies to mouse NK1.1 (PK136), mouse PVRIG (Clone 1) and human PVRIG (Clone 2) were purified in-house from hybridoma cell supernatant. Anti-PD-L1 antibody (10F.9G2) and anti-CD8β antibody (53-5.8) were purchased from Bio X Cell (Lebanon, USA). The isotype-matched control antibodies (rat IgG) were purified in-house from rat serum. The following reagents were used: FITC-conjugated antibodies to mouse CD226 (10E5, BioLegend, San Diego, USA), CD69 (H1.2F3, BD Pharmingen, San Diego, USA) and CD107a (1D4B, BD Pharmingen, San Diego, USA); PE-conjugated antibodies to mouse CD8β (H35-17.2, BD Pharmingen, San Diego, USA), CD96 (3.3, BioLegend, San Diego, USA), NKG2D (CX5, BD Pharmingen, San Diego, USA) and Perforin (eBioOMAK-D, eBioscience, San Diego, USA); PerCP-eFluor 710-conjugated antibody to mouse Granzyme B (NGZB, eBioscience, San Diego, USA); PE-Cy7-conjugated antibodies to mouse TIGIT (GIGD7, eBioscience, San Diego, USA), TRAIL (N2B2, BioLegend, San Diego, USA) and Ki67(SolA15, eBioscience, San Diego, USA); BV421-conjugated antibiodies to mouse Tim-3 (RMT3-23, BioLegend, San Diego, USA), TNF-α (MP6-XT22, BioLegend, San Diego, USA) and FasL (MFL3, eBioscience, San Diego, USA); BV510-conjugated antibody to mouse CD45 (30-F11, BD Pharmingen, San Diego, USA); BV605-conjugated antibody to mouse NK1.1 (PK136, BioLegend, San Diego, USA); Alexa fluor 647-conjugated antibody to mouse NKp46 (29A1.4, BD Pharmingen, San Diego, USA); BV785-conjugated antibody to mouse PD-1 (29F.1A12, BioLegend, San Diego, USA); BV786-conjugated antibody to mouse NKG2A/C/E (20d5, BD Pharmingen, San Diego, USA) and IFN-γ (XMG1.2, BD Pharmingen, San Diego, USA); BUV395-conjugated antibody to mouse CD3ε (145-2C11, BD Pharmingen, San Diego, USA); BUV563-conjugated antibody to mouse CD4 (GK1.5, BD Pharmingen, San Diego, USA); BUV737-conjugated antibody to mouse CD8α (53-6.7, BD Pharmingen, San Diego, USA).

The following antibodies were also used: FITC-conjugated antibody to human IFN-γ (B27, BioLegend, San Diego, USA); PE-conjugated antibody to human TNF-α (Mab11, BioLegend, San Diego, USA); PerCP-Cy5.5-conjugated antibody to human CD3ε (HIT3a, BioLegend, San Diego, USA); PE-Cy7-conjugated antibody to human CD8α (RPA-T8, BD Pharmingen, San Diego, USA); BV421-conjugated antibody to human CD4 (RPA-T4, BD Pharmingen, San Diego, USA); BV510-conjugated antibody to human CD16 (3G8, BD Pharmingen, San Diego, USA) and CD107a (H4A3, BioLegend, San Diego, USA); BV605-conjugated antibody to human CD56 (5.1H11, BioLegend, San Diego, USA); and APC-conjugated antibody to human PVRIG (W16216D, BioLegend, San Diego, USA).

### Intracellular cytokine staining

For CD107a and intracellular cytokine staining, splenocytes and TILs cells were stimulated for 4 h with 30 ng/mL phorbol 12-myristate 13-acetate (PMA, Sigma-Aldrich, St. Louis, USA) and 1 µM ionomycin (Sigma-Aldrich, St. Louis, USA) in the presence of 2.5 μg/mL monensin (eBioscience, San Diego, USA). After stimulation, cells were stained for surface markers, fixed and permeabilized with FoxP3 fixation buffer (eBioscience, San Diego, USA) according to the manufacturer’s instructions. Fixed cells were stained with antibodies to IFN-γ, TNF-α and Granzyme B. All samples were acquired on an LSRFortessa (BD, Franklin Lakes, USA) and were analyzed using FlowJo software (BD, Franklin Lakes, USA).

### In vivo cell depletion

For depletion of NK1.1^+^ cells or CD8^+^ T cells, mice were given intraperitoneal injection of 200 μg mAb to NK1.1 (PK136; purified in-house from cell supernatant) or 200 μg mAb to CD8β (53-5.8, Bio X Cell, Lebanon, USA) 24 h before challenge, and then the antibodies were injected once every week.

### Isolation of human PBMCs and NK cells

Peripheral blood samples from healthy controls were collected from The First Affiliated Hospital of Anhui Medical University (Hefei, China). And peripheral blood mononuclear cells (PBMCs) were isolated by Ficoll-Paque (GE Healthcare, Little Chalfont, UK) density gradient centrifugation according to the manufacturer’s instructions. NK cells were purified by negative selection using human NK Cell Isolation Kit (Miltenyi Biotec, Bergisch Gladbach, Germany) according to manufacturer’s instructions. The purity of NK cells was > 90% as determined by flow cytometry. Human PBMCs and purified NK cells were cultured in RPMI1640 medium with 10% FBS and 100 IU/mL recombinant human IL-2.

### In vitro co-culturing system

1 × 10^6^ human PBMCs or 1 × 10^5^ NKG cells were co-cultured with 4 × 10^4^ SW620 colon cancer cells for 24 h either in the presence of anti-human PVRIG antibody or isotype-matched control antibody (mouse IgG1). CD107a antibody and monensin were added to the culture 4 h before harvest.

### Cytotoxicity assay

Tumor cell lines were labeled with CFSE (Thermo Fisher Scientific, Waltham, USA) according to the manufacturer’s instructions. Labeled tumor cells were co-cultured with effector cells in 96-well plate for 4 h in the presence of anti-human PVRIG antibody or isotype-matched control antibody (mouse IgG1) at different effector/target ratios. For the spontaneous death control, CFSE-labeled target cells were cultured alone followed by the addition of 7AAD, and lysed cells (CFSE^+^ 7AAD^+^) were identified by flow cytometry.

### Immunohistochemistry

Paraffin sections of human tumor tissues were purchased from the Shanghai Outdo Biotech Co. Ltd. (Shanghai, China). Paraffin sections were de-waxed, rehydrated, subjected to heat-induced epitope retrieval (HIER) and followed by incubation with primary antibodies to human PVRIG (Clone 2, generated in house) and PVRL2 (AF2229, R&D Systems, Minneapolis, USA) respectively. The signal was detected using the DAB Peroxidase Substrate Kit (SK-4100, Vector Labs, Burlingame, USA).

### TCGA data analysis

The Cancer Genome Atlas (TCGA) database was used to analyze the gene expression correlations between *PVRIG* and other immune checkpoints, as well as the overall survival of colon adenocarcinoma patients based on their *PVRIG* gene expression. The correlations were analyzed by UCSC xena at https://xena.ucsc.edu. The Kaplan–Meier survival of colon adenocarcinoma patients was analyzed by OncoLnc at http://www.oncolnc.org.

### Statistical analysis

Statistical analyses were performed in GraphPad Prism (La Jolla, USA) using appropriate tests as indicated in the figure legends (unpaired two-tailed *t* test, paired two-tailed *t* test, one-way ANOVA followed by Tukey’s multiple comparisons test, two-way ANOVA or the Mantel–Cox test). *P* < 0.05 was considered significant in all analyses.

## Results

### High expression of PVRIG is associated with NK cell exhaustion

**Fig. 1:**
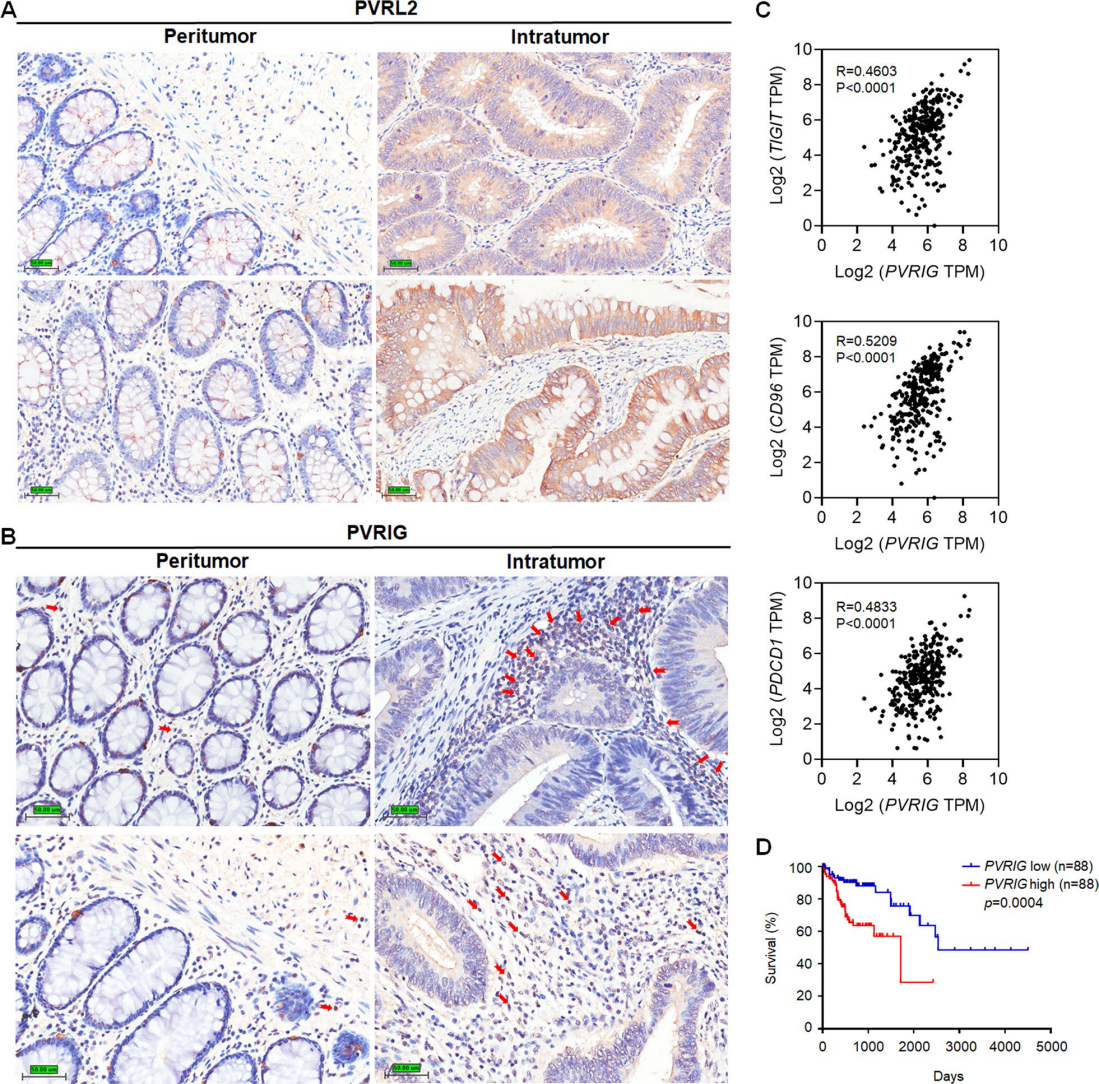
PVRIG is mainly expressed on lymphocytes and associated with poor clinical outcomes in patients with colon adenocarcinoma. **a** Representative immunohistochemical staining of PVRL2 in the peritumoral and intratumoral tissues of colon adenocarcinoma (COAD) patients. Original magnifications: 20×. Bar = 50 μm. **b** Representative immunohistochemical staining of PVRIG in the peritumoral and intratumoral tissues of COAD patients. Positive staining is indicated by red arrows. Original magnifications: 20X. Bar = 50 μm. **c** Correlation between *PVRIG* and other immune checkpoint receptor (*TIGIT*, *CD96* or *PDCD1*) based on the gene expression analysis of COAD patients (*n* = 329 per group, TCGA database). **d** Kaplan–Meier survival of COAD patients (*n* = 88 per group, cutoff: 20–20, TCGA database) based on the level of *PVRIG* mRNA expression (*PVRIG* low, blue; *PVRIG* high, red). Data were analyzed for significance by the log-rank test

Nectin and nectin-like families are important adhesion molecules and are overexpressed in various types of tumors [13, 33–35]. Consistent with previous studies, we found that PVRL2 was highly expressed on tumor cells in colon adenocarcinoma (COAD) patients while barely expressed on peritumoral mucosa tissues (Fig. 1a). PVRIG, a new immune checkpoint identified on T cells, binds to its ligand PVRL2 with higher affinity than TIGIT and CD226 [24]. PVRIG was reported to express on immune cells [24]; in our study, we also confirmed the expression of PVRIG on immune cells in COAD patients, as indicated by the red arrows (Fig. 1b). The upregulation of PVRL2 in tumor tissues may be the main reason for the over-expressed PVRIG observed in TILs. Analysis of TCGA database showed that gene expression of *PVRIG* in COAD patients is positively correlated to the gene expression of other immune checkpoint receptors, including *TIGIT*, *CD96* and *PDCD1* (Fig. 1c). Furthermore, COAD patients were divided into two groups (*PVRIG* high and *PVRIG* low) based on their intratumoral *PVRIG* gene expression as reported by the TCGA database, not surprisingly, patients with high *PVRIG* expression exhibited shorter overall survival compared to those with low *PVRIG* expression across multiple cutoff values (Fig. 1d, Additional file 1: Fig. S1), suggesting the potential of PVRIG as a prognostic marker in predicting the outcomes of COAD patients.

**Fig. 2:**
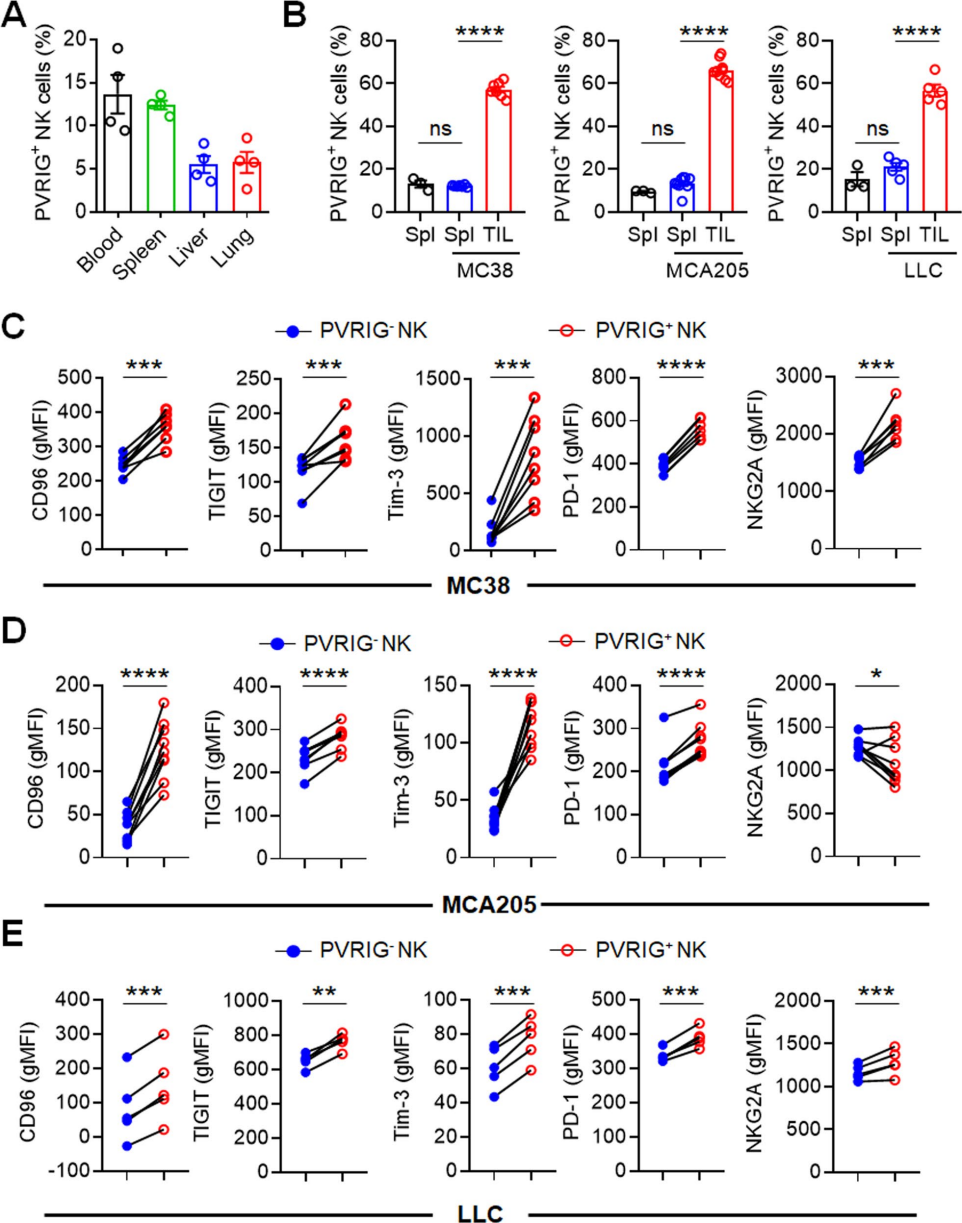
PVRIG expression is associated with exhausted phenotype of tumor-infiltrating NK cells. **a** Percentage of PVRIG^+^ NK cells in PBMCs (blood), splenocytes (spleen), liver MNCs (liver) and lung MNCs (lung) from normal B6 mice (*n* = 4). **b** Percentage of PVRIG^+^ NK cells in splenocytes (Spl) and tumor-infiltrating lymphocytes (TIL) from WT mice (*n* = 3) or tumor-bearing mice subcutaneously injected with either 2 × 10^5^ MC38 (*n* = 8), 2 × 10^5^ MCA205 (*n* = 9) or 1 × 10^6^ LLC (*n* = 5) cells, analyzed when tumor size reaches 300 mm^3^. **c** Geometric MFI of CD96, TIGIT, Tim-3, PD-1 or NKG2A in tumor-infiltrating PVRIG^−^ NK cells (PVRIG^−^ NK) and PVRIG^+^ NK cells (PVRIG^+^ NK) from MC38-bearing mice (*n* = 8 for CD96, TIGIT, Tim-3 and NKG2A; *n* = 7 for PD-1). **d** Geometric MFI of CD96, TIGIT, Tim-3, PD-1 or NKG2A in tumor-infiltrating PVRIG^−^ NK cells (PVRIG^−^ NK) and PVRIG^+^ NK cells (PVRIG^+^ NK) from MCA205-bearing mice (*n* = 9). **e** Geometric MFI of CD96, TIGIT, Tim-3, PD-1 or NKG2A in tumor-infiltrating PVRIG^−^ NK cells (PVRIG^−^ NK) and PVRIG^+^ NK cells (PVRIG^+^ NK) from LLC-bearing mice (*n* = 5). Each symbol represents an individual mouse (**a**–**e**), lines connect values for the same mouse (**c**–**e**). Data were representative of at least two independent experiments. Error bars represent means ± s.e.m. Statistical significance was determined using paired two-tailed *t* test (**c**–**e**) or one-way ANOVA followed by Tukey’s multiple-comparisons test (**b**). ns, not significant (*p* > 0.05); *p < 0.05; **p < 0.01; ***p < 0.001 and ****p < 0.0001

Previous study has reported positive detection of PVRIG on both T cells and NK cells from healthy peripheral blood and various human cancer tissues [24, 36]; however, the expression of PVRIG on murine NK cells has not been fully revealed. We found that in normal mice, only 5–15% of NK cells expressed PVRIG in the peripheral blood, spleen, liver and lung (Fig. 2a). However, the percentage of PVRIG^+^ NK cells among TILs was significantly higher in three murine models of subcutaneously administered tumors (MC38 colon cancer, MCA205 fibrosarcoma and LLC lung cancer) compared with those in the spleen of normal mice and tumor-bearing mice (*P* < 0.0001; Fig. 2b). PVRIG was reported to be involved in the exhaustion of CD8^+^ T cells during tumor progression [26]. To illustrate the effect of PVRIG on NK cells in tumor microenvironment, we compared PVRIG^+^ and PVRIG^−^ tumor-infiltrating NK cells from three murine tumor models (MC38 colon cancer, MCA205 fibrosarcoma and LLC lung cancer). We found that tumor-infiltrating PVRIG^+^ NK cells expressed higher level of inhibitory receptors, many of which are also exhaustion markers, including CD96, TIGIT, Tim-3, PD-1 and NKG2A compared with PVRIG^−^ tumor-infiltrating NK cells (Fig. 2c–e), suggesting a more exhausted phenotype of tumor-infiltrating PVRIG^+^ NK cells.

### PVRIG deficiency prevents NK cell exhaustion and slows tumor growth in tumor-bearing mice

**Fig. 3:**
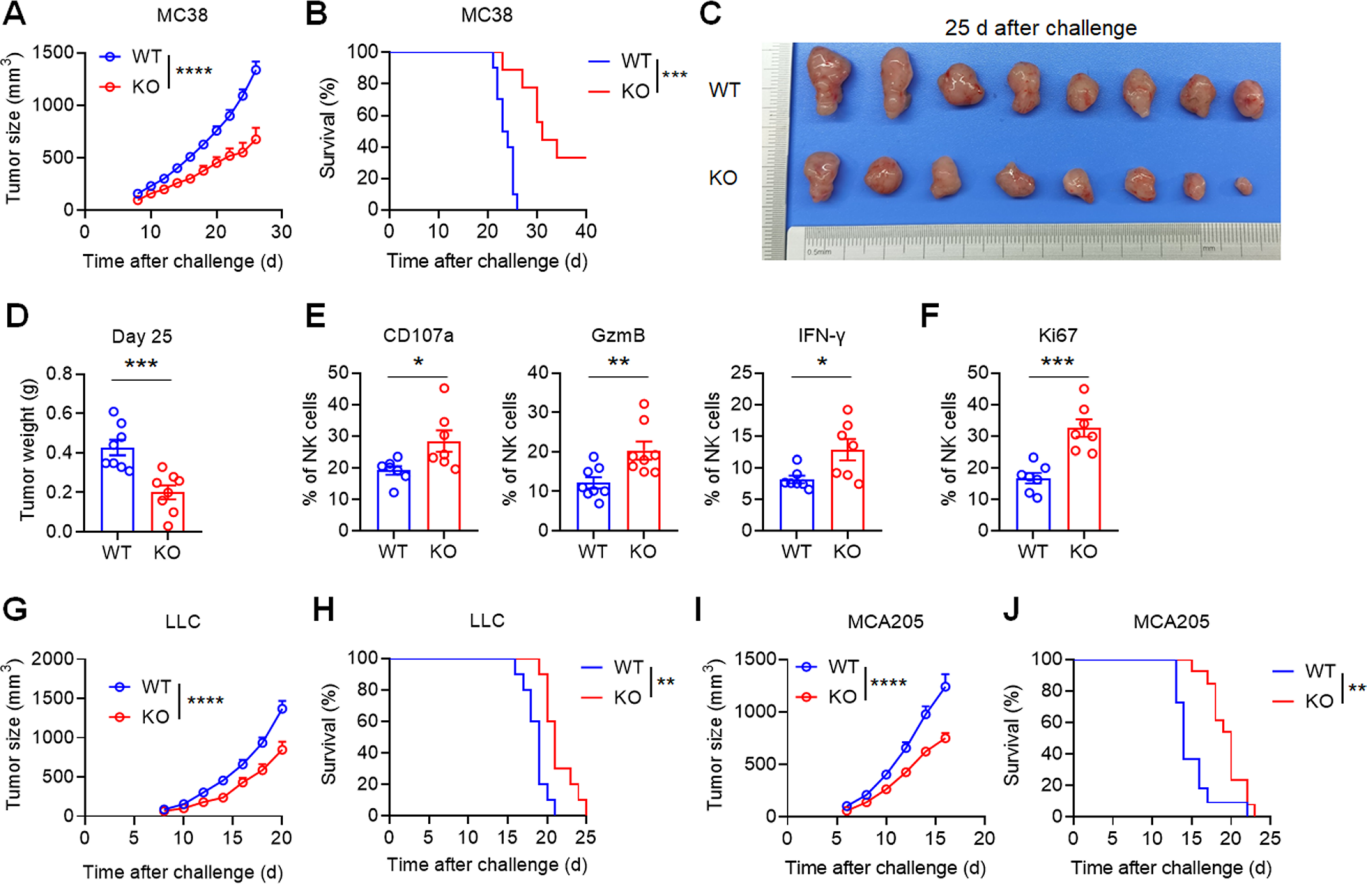
PVRIG deficiency slows tumor growth and prevents exhaustion of tumor-infiltrating NK cells in various solid tumor models. **a**, **b** WT (*n* = 10) and PVRIG KO (*n* = 9) mice were subcutaneously injected with 2 × 10^5^ MC38 cells, and the tumor size was measured every two days. **a** Median tumor size and **b** mouse survival over time were shown. **c** Representative photograph and **d** weight of tumor as in **a** (*n* = 8 per group) on day 25 after challenge. **e** Percentage of CD107a^+^, Granzyme B (GzmB)^+^ or IFN-γ^+^ tumor-infiltrating NK cells in WT and PVRIG KO mice (*n* = 7 or 8 per group). **f** Percentage of Ki67^+^ tumor-infiltrating NK cells in WT and PVRIG KO mice (*n* = 7 per group). **g**, **h** WT (*n* = 10) and PVRIG KO (*n* = 10) mice were subcutaneously injected with 1 × 10^6^ LLC cells and the tumor size was measured every two days. **g** Median tumor size and **h** mouse survival over time were shown. **i**, **j** WT (*n* = 11) and PVRIG KO (*n* = 13) mice were subcutaneously injected with 5 × 10^4^ MCA205 cells and the tumor size was measured every 2 days. **i** Median tumor size and **j** mouse survival over time were shown. Each symbol represents an individual mouse. Data were representative of at least two independent experiments. Error bars represent means ± s.e.m. Statistical significance was determined using two-way ANNOVA (**a**, **g**, **i**), Mantel–Cox test (**b**, **h**, **j**) or unpaired two-tailed *t* test (**d**–**f**). *p < 0.05; **p < 0.01; ***p < 0.001 and ****p < 0.0001

To evaluate the importance of PVRIG during tumor progression, we generated PVRIG-deficient mice (Additional file 1: Fig. S2). We compared the splenic NK cell frequency (Additional file 1: Fig. S3A), the developmental NK cell subsets (Additional file 1: Fig. S3B), the expression of activating receptors and inhibitory receptors (Additional file 1: Fig. S3C, D) and the expression of effector molecules (Additional file 1: Fig. S3E, F) between normal mice and PVRIG-deficient mice and confirmed no differences in the phenotypical and functional features between these two mouse strains under steady state. Next, we subcutaneously administered tumor cells (MC38, MCA205 and LLC) to normal mice and PVRIG-deficient mice to investigate the effect of PVRIG on tumor growth and the survival of tumor-bearing mice. In MC38 colon cancer model, PVRIG-deficient mice showed significantly slower tumor growth (*P* < 0.0001) and longer survival time (*P* < 0.001) compared with wild-type mice (Fig. 3a, b). Mice were killed on day 25 after challenge and tumors were photographed and weighed. Smaller tumors and lighter weight of tumors (*P* < 0.001) were observed in PVRIG-deficient mice (Fig. 3c, d). Higher percentage of CD107a^+^, GzmB^+^ and IFN-γ^+^ tumor-infiltrating NK cells were observed respectively in PVRIG-deficient mice (Fig. 3e). Furthermore, the proliferation of tumor-infiltrating NK cells was enhanced in PVRIG-deficient mice as indicated by the Ki67 expression (*P* < 0.001; Fig. 3f). Previous study has shown that CD8^+^ T cells from PVRIG-deficient mouse exhibited a stronger antigen-specific effector response during acute *Listeria monocytogenes* infection and MC38 syngeneic model [26]. Our study showed higher expression of Ki67, CD107a, GzmB and IFN-γ in the tumor-infiltrating CD8^+^ T cells from PVRIG-deficient mice (Additional file 1: Fig. S4A–C), suggesting that besides NK cells, CD8^+^ T cells in PVRIG-deficient mice were also more functional. Consistent with MC38 model, significant smaller tumors (*P* < 0.0001) and longer survival (*P* < 0.01) were observed in PVRIG-deficient mice subcutaneously injected with LLC cells or MCA205 cells (Fig. 3g–j). Taken together, PVRIG deficiency slowed tumor growth and prolonged survival of tumor-bearing mice, further, tumor-infiltrating NK cells from PVRIG-deficient mice were potentially more functional and proliferative than those in wild-type mice, suggesting that PVRIG deficiency could possibly prevent the exhaustion of tumor-infiltrating NK cells.

### PVRIG blockade reverses NK cell exhaustion and inhibits tumor growth in tumor-bearing mice

**Fig. 4:**
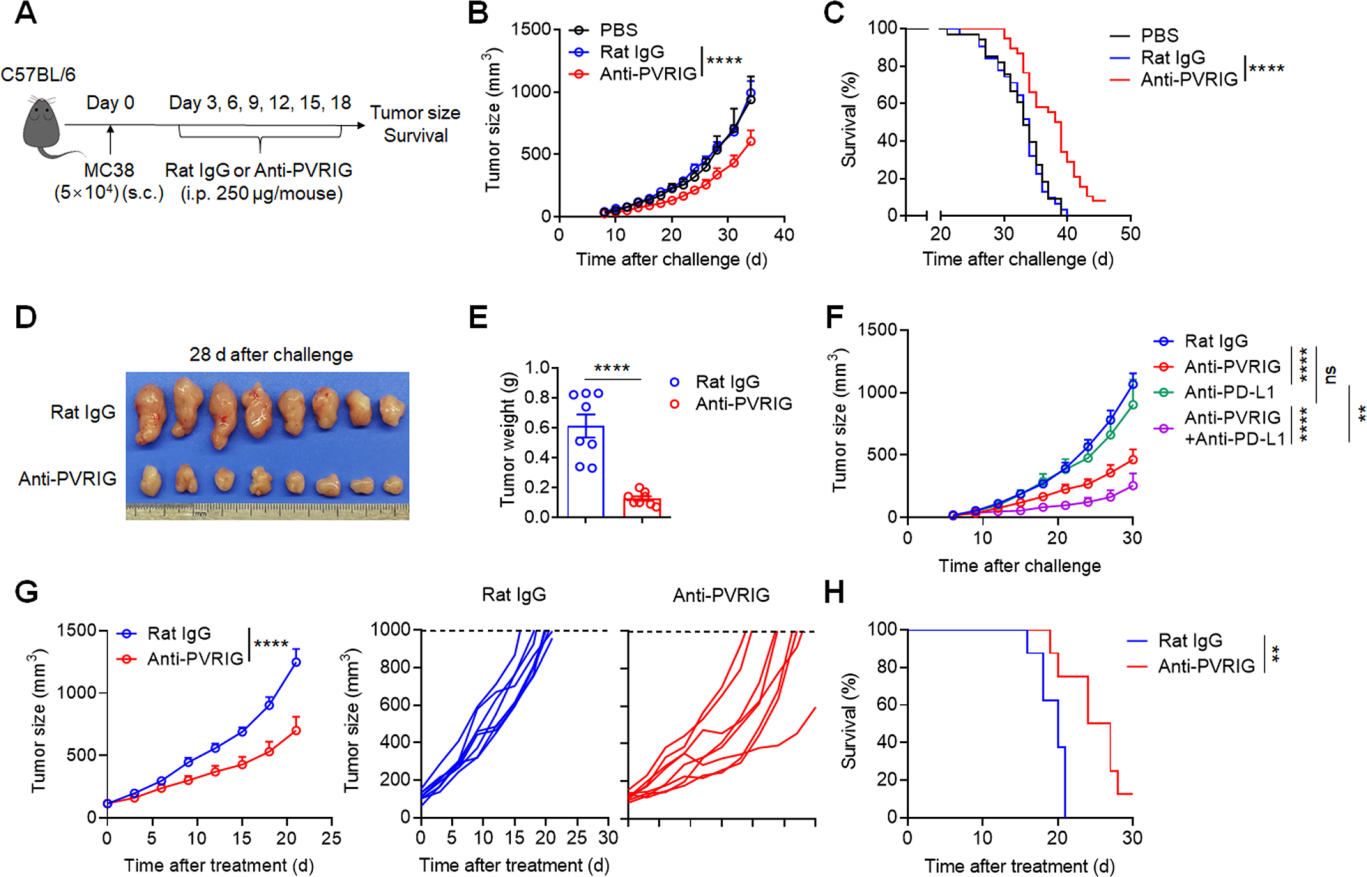
Both early and late blockades of PVRIG inhibit tumor growth in MC38 tumor-bearing mice. **a** Experimental protocol for murine colon cancer model used in **b**–**e**. Mice were injected with PBS, anti-PVRIG mAb or isotype-matched control mAb (rat IgG) intraperitoneally (i.p.) at various time points after injection of 5 × 10^4^ MC38 tumor cells subcutaneously (s.c.) on day 0. **b** Median tumor size (*n* = 10 (PBS), *n* = 8 (rat IgG), *n* = 12 (anti-PVRIG)) measured at each time point. **c** Overall survival of mice treated with PBS (*n* = 33), Rat IgG (*n* = 31) or anti-PVRIG mAb (*n* = 38). **d** Representative photograph and **e** weight of tumor (*n* = 8 per group) on day 28 after challenge. **f** C57BL/6 mice were inoculated subcutaneously (s.c.) with 5 × 10^4^ MC38 colon cancer cells on day 0. Mice were grouped randomly on day 3. And then mice were injected with isotype-matched control mAb (Rat IgG), anti-PVRIG mAb, anti-PD-L1 mAb or anti-PVRIG + anti-PD-L1 mAb intraperitoneally (i.p.) starting on day 3 for six times (*n* = 8 per group). And median tumor size was shown. **g**, **h** C57BL/6 mice were inoculated subcutaneously (s.c.) with 2 × 10^5^ MC38 colon cancer cells. Mice were grouped randomly when tumor size reaches around 100–150 mm^3^ and treated with isotype-matched control mAb (Rat IgG) or anti-PVRIG mAb intraperitoneally (i.p.) for six times (*n* = 8 per group). **g** Median (left) and individual (right) tumor size was shown. **h** Overall survival of tumor-bearing mice was shown. Each symbol represents an individual mouse. Data were representative of at least two independent experiments. Error bars represent means ± s.e.m. Statistical significance was determined using two-way ANNOVA (**b**, **f**, **g**), Mantel–Cox test (**c**, **h**) or unpaired two-tailed *t* test (**e**). ns, *p* > 0.05; **p < 0.01; ***p < 0.001 and ****p < 0.0001

To assess the effect of PVRIG blockade in vivo, we generated rat anti-mouse PVRIG mAb (Clone 1) and verified its function of blocking PVRIG-PVRL2 interaction (Additional file 1: Fig. S5A–E). Mice were subcutaneously inoculated with MC38 tumor cells to generate murine colon tumor models, followed by treatment with anti-PVRIG mAb, rat IgG, or PBS on day 3 post-tumor graft (tumor size was around 10 mm^3^) (Fig. 4a). The results indicated that early treatment with anti-PVRIG mAb significantly inhibited tumor growth (*P* < 0.0001; Fig. 4b) and extended the overall survival (*P* < 0.0001; Fig. 4c) of tumor-bearing mice compared with the control groups. Smaller tumor size and lower tumor weight (*P* < 0.001) were also observed on day 28 after tumor challenge in mice treated with anti-PVRIG mAb (Fig. 4d, e). In addition, we also evaluated the combined therapeutic value of anti-PVRIG and anti-PD-L1. Although anti-PD-L1 mAb alone was not effective, combined blockade with anti-PVRIG mAb showed impressive therapeutic effects. More importantly, combinatorial treatment showed better therapeutic effects than using either mAb alone (Fig. 4f), suggesting the prospect of combining anti-PVRIG and anti-PD-L1 in clinical applications. Early treatment with anti-PVRIG mAb showed impressive outcome; besides, late treatment is also an important indicator to evaluate the therapeutic value of checkpoint immunotherapy. Therefore, in addition to the early treatment, we also treated mice with anti-PVRIG mAb when MC38 tumor size reaches 100–150 mm^3^. The results indicated that late treatment with anti-PVRIG mAb significantly inhibited tumor growth (*P* < 0.0001; Fig. 4g) and prolonged the overall survival of tumor-bearing mice (*P* < 0.01; Fig. 4h). These results indicated that both early and late treatments of anti-PVRIG mAb were effective in reducing tumor growth, again, implying the importance and therapeutic value of anti-PVRIG mAb in clinical applications.

**Fig. 5:**
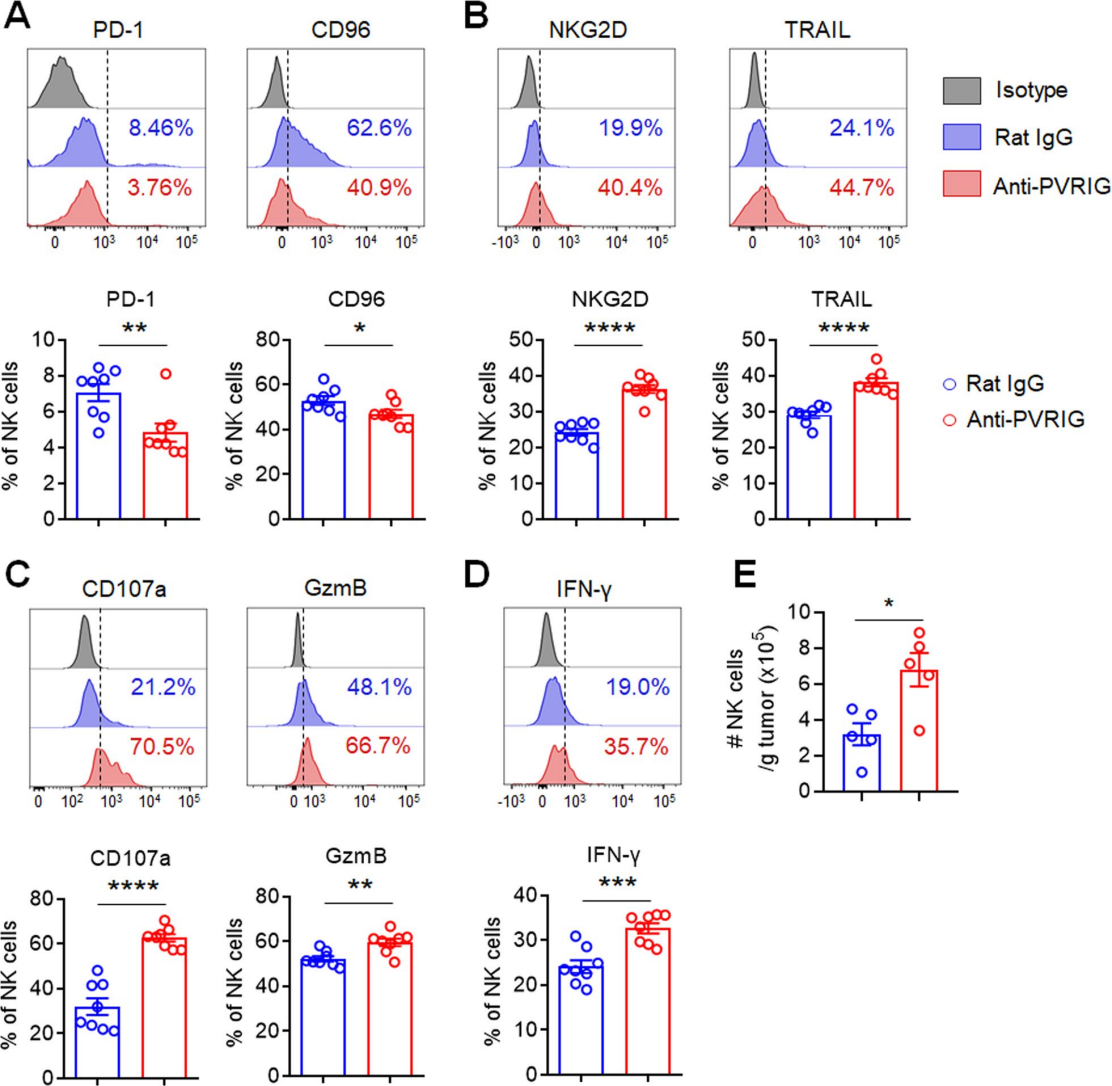
Blockade of PVRIG reverses exhaustion of tumor-infiltrating NK cells in tumor-bearing mice. Mice were injected with PBS, anti-PVRIG mAb or isotype-matched control mAb (rat IgG) intraperitoneally (i.p.) at various time points after injection of 5 × 10^4^ MC38 tumor cells subcutaneously (s.c.) on day 0 and killed on day 28 after challenge as described in Fig. 4a (*n* = 8 per group). **a** Representative histograms (top) and quantification (bottom) of PD-1 and CD96 expression in tumor-infiltrating NK cells. **b** Representative histograms (top) and quantification (bottom) of NKG2D and TRAIL expression in tumor-infiltrating NK cells. **c** Representative histograms (top) and quantification (bottom) of CD107a and Granzyme B (GzmB) expression in tumor-infiltrating NK cells. **d** Representative histograms (top) and quantification (bottom) of IFN-γ synthesis in tumor-infiltrating NK cells. **e** Absolute number of tumor-infiltrating NK cells in mice treated with rat IgG or anti-PVRIG mAb. Each symbol represents an individual mouse (*n* = 8 (**a**–**d**) or *n* = 5 (**e**) per group). Data were representative of two independent experiments. Error bars represent means ± s.e.m. Statistical significance was determined using unpaired two-tailed *t* test. *p < 0.05; **p < 0.01; ***p < 0.001 and ****p < 0.0001

To explore the possible mechanisms underlying the therapeutic effects of anti-PVRIG mAb treatment, we compared the functional status of TILs in mice treated with anti-PVRIG mAb and those with control antibody. Tumor-bearing mice treated with anti-PVRIG mAb showed lower expression of inhibitory receptors CD96 and PD-1 (Fig. 5a) and higher expression of activating receptor NKG2D and cytotoxic molecule TRAIL on tumor-infiltrating NK cells (Fig. 5b). Furthermore, use of anti-PVRIG mAb also enhanced the cytotoxic potential of tumor-infiltrating NK cells, indicated by higher expression of CD107a and GzmB (Fig. 5c). Besides, IFN-γ secretion was significantly improved in tumor-bearing mice treated with anti-PVRIG mAb compared with those treated with rat IgG (Fig. 5d). In addition, tumor-bearing mice treated with anti-PVRIG mAb showed lower frequency of tumor-infiltrating CD8^+^ T cells expressing inhibitory receptor CD96 (Additional file 1: Fig. S6A) and higher frequency of tumor-infiltrating CD8^+^ T cells expressing NKG2D, TRAIL (Additional file 1: Fig. S6B), CD107a, GzmB (Additional file 1: Fig. S6C) and IFN-γ (Additional file 1: Fig. S6D). More importantly, anti-PVRIG treatment also increased the number of NK cells and CD8^+^ T cells infiltrated into the tumor (Fig. 5e, Additional file 1: Fig. S6E). Collectively, these results demonstrated that therapeutically blocking PVRIG in vivo elicited potent systemic anti-tumor immunity against established tumors and inhibited tumor growth possibly by reversing the exhaustion of tumor-infiltrating NK cells as well as CTLs.

### Both NK cells and CD8^+^ T cells contribute to the anti-tumor efficacy of PVRIG blockade

**Fig. 6:**
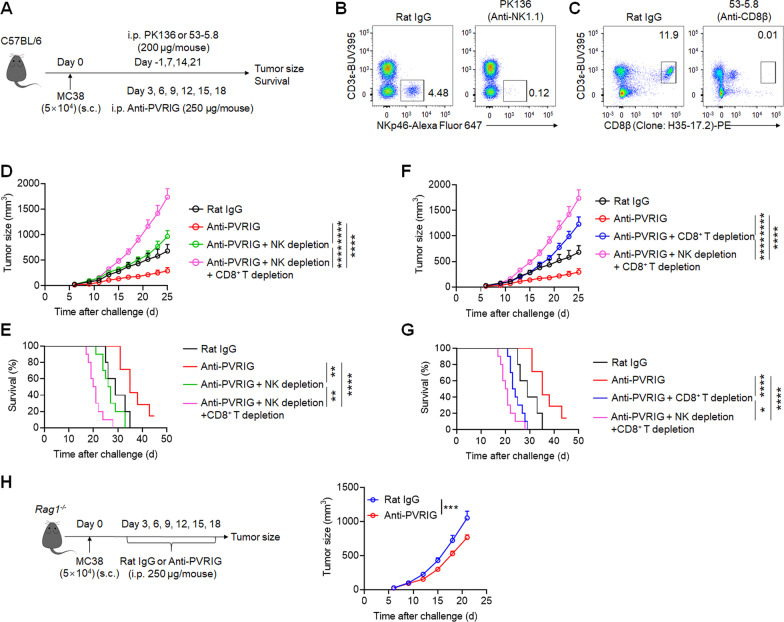
Both NK cells and CD8^+^ T cells contribute to the anti-tumor efficacy of PVRIG blockade. **a** Experimental protocol for murine colon cancer model used in **d**–**g**. Mice were injected with isotype-matched control mAb (rat IgG), anti-PVRIG mAb, anti-PVRIG mAb combined with PK136 (anti-NK1.1), anti-PVRIG mAb combined with 53-5.8 (anti-CD8β) or anti-PVRIG mAb combined with PK136 and 53-5.8 intraperitoneally (i.p.) at various time points after injection of 5 × 10^4^ MC38 tumor cells subcutaneously (s.c.) on day 0. **b** Representative flow plots of splenic NK cells in mice treated with PK136 (anti-NK1.1) antibody or control antibody. **c** Representative flow plots of splenic CD8^+^ T cells in mice treated with 53-5.8 (anti-CD8β) antibody or control antibody. **d** Median tumor size measured at various time points and **e** overall survival of mice with different treatments (*n* = 5 (Rat IgG), *n* = 7 (anti-PVRIG), *n* = 10 (anti-PVRIG + NK depletion), *n* = 10 (anti-PVRIG + NK depletion + CD8^+^ T depletion)). **f** Median tumor size measured at various time points and **g** overall survival of mice with different treatments (*n* = 5 (Rat IgG), *n* = 7 (anti-PVRIG), *n* = 10 (anti-PVRIG + CD8^+^ T depletion), *n* = 10 (anti-PVRIG + NK depletion + CD8^+^ T depletion)). **h** *Rag1*^*−/−*^ mice were injected with isotype-matched control mAb (rat IgG) or anti-PVRIG mAb intraperitoneally (i.p.) at various time points after injection of 5 × 10^4^ MC38 tumor cells subcutaneously (s.c.) on day 0. And median tumor size was shown on the right. Data are representative of two independent experiments. Error bars represent means ± s.e.m. Statistical significance was determined using two-way ANNOVA (**d**, **f**, **h**) or Mantel–Cox test (**e**, **g**). *p < 0.05; **p < 0.01; ***p < 0.001; ****p < 0.0001

Blockade of PVRIG reversed the exhaustion of both NK cells and CD8^+^ T cells; to further investigate the roles of NK cells and CD8^+^ T cells in the therapeutic blockade of PVRIG, we depleted NK cells or/and CD8^+^ T cells in tumor-bearing mice by treating them with depleting antibodies against NK cell receptor NK1.1 (PK136) and CD8β (53-5.8), respectively (Fig. 6a). Successful depletion of NK cells or CD8^+^ T cells was verified by flow cytometric analysis (Fig. 6b, c). Consistent with our previous results (here as positive control), anti-PVRIG mAb significantly inhibited tumor growth (*P* < 0.0001) and prolonged overall survival (*P* < 0.0001) of tumor-bearing mice (Fig. 6d–g). However, along with anti-PVRIG mAb treatment, depletion of NK cells significantly accelerated tumor growth and shortened the overall survival of tumor-bearing mice (Fig. 6d, e), meanwhile, depletion of CD8^+^ T cells resulted in a very similar fashion (Fig. 6f, g), suggesting that the presence of NK cells and CTLs both contributes to the therapeutic efficacy of anti-PVRIG mAb. As expected, concurrent depletion of NK and CD8^+^ T cells resulted in the fastest tumor growth and shortest survival time in tumor-bearing mice (Fig. 6d–g). To further asses the importance of NK cells in PVRIG blockade, we used *Rag1*^*−/−*^ mice (which lack both T cells and B cells) to construct the MC38 subcutaneous tumor model. The results showed that the use of anti-PVRIG mAb significantly inhibited tumor growth in *Rag1*^*−/−*^ mice compared to those treated with control antibody (*P* < 0.001; Fig. 6h), suggesting that anti-PVRIG mAb was effective even in the absence of adaptive immunity. Overall, these results indicated that although NK cells and CTLs both contributed to the therapeutic outcome of PVRIG blockade, anti-PVRIG mAb could also be effective in the absence of adaptive immunity, again, suggesting the importance of NK cells in PVRIG-targeted treatment.

### Blockade of human PVRIG enhances NK cell cytotoxicity and inhibits tumor growth in NK cell- or PBMC-reconstituted xenograft mice

**Fig. 7:**
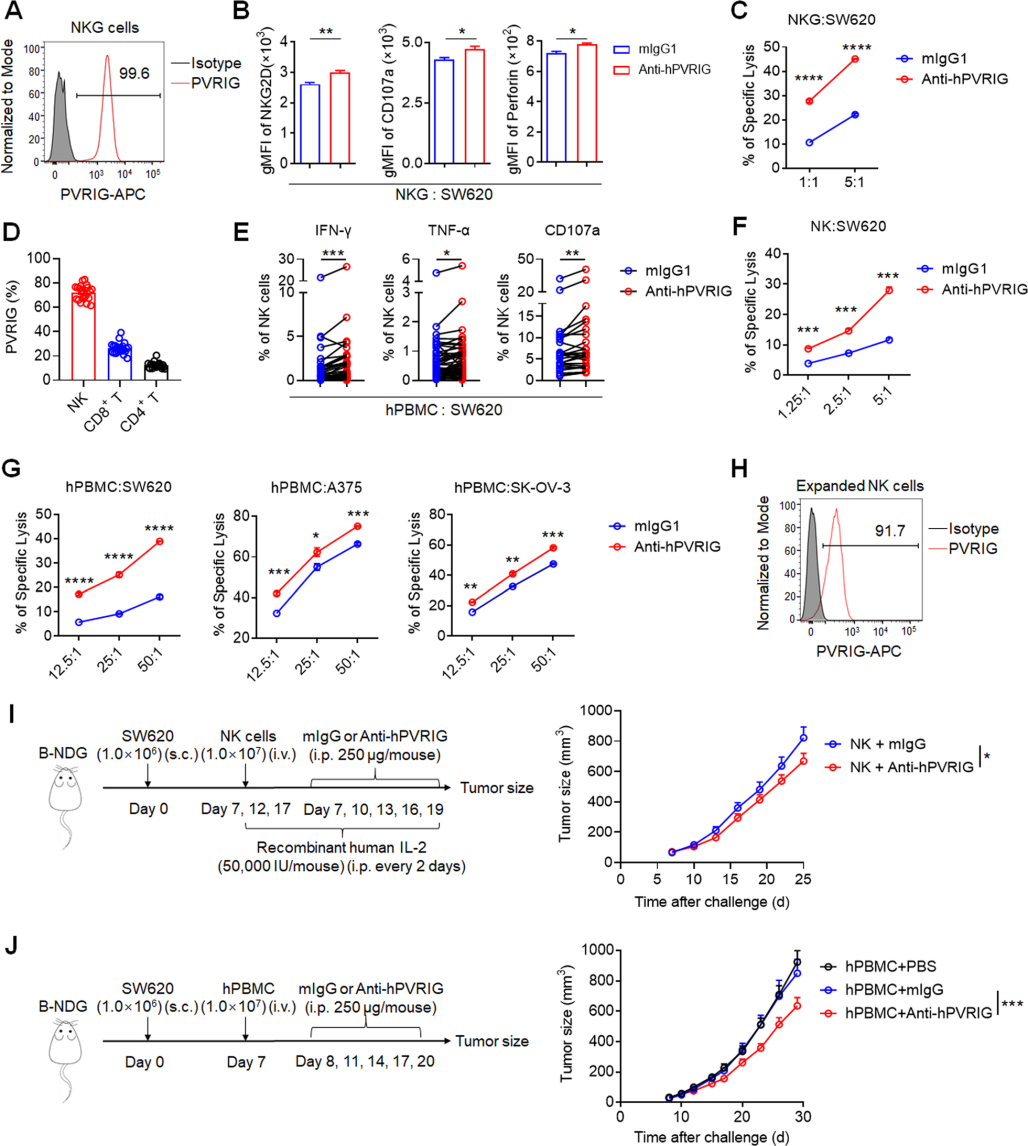
Blockade of PVRIG enhances human NK cell cytotoxicity and inhibits tumor growth in NK cell- or PBMC-reconstituted xenograft mice. **a** Representative histogram of PVRIG expression in human NKG cell line. **b** NKG cells were co-cultured with SW620 cells at 2.5:1 ratio for 24 h in the presence of anti-hPVRIG antibody or mIgG1 control antibody. The expression of NKG2D, CD107a and perforin in NKG cells was analyzed by flow cytometry. **c** Cytotoxicity of NKG cells against human colon cancer cell line SW620 in the presence of anti-hPVRIG antibody (red) or mIgG1 control antibody (blue) at E/T ratios of 1:1 and 5:1. **d** Flow cytometric analysis of PVRIG expression in CD56^+^ NK cells, CD8^+^ T cells and CD4^+^ T cells from healthy human PBMCs (*n* = 21). **e** Human PBMCs were co-cultured with SW620 cells at an E/T ratio of 25:1 for 24 h in the presence of anti-hPVRIG antibody or mIgG1 control antibody. The expression of IFN-γ (*n* = 47), TNF-α (*n* = 47) and CD107a (*n* = 26) in NK cells was analyzed by flow cytometry. **f** Cytotoxicity of purified human NK cells against SW620 cells in the presence of anti-hPVRIG antibody or mIgG1 control antibody at indicated E/T ratios. **g** Cytotoxicity of human PBMCs against SW620, A375 and SK-OV-3 tumor cells in the presence of anti-hPVRIG antibody or mIgG1 control antibody at various E/T ratios was analyzed, respectively. **h** Representative histogram of PVRIG expression in expanded human NK cells. **i** B-NDG mice were inoculated subcutaneously with SW620 colon cancer cells on day 0. Mice were grouped randomly and expanded NK cells were injected intravenously (i.v.) on days 7, 12 and 17. Mice were then treated with anti-hPVRIG mAb (*n* = 9) or isotype-matched control mAb (mouse IgG) (*n* = 9) intraperitoneally (i.p.) every three days starting on day 7 for five times. All mice were injected intraperitoneally with 50,000 IU recombinant human IL-2 every two days starting on day 7. And median tumor size was shown on the right. **j** B-NDG mice were inoculated subcutaneously with SW620 colon cancer cells on day 0. Mice were grouped randomly and human PBMCs were injected intravenously (i.v.) on day 7. Mice were then treated with PBS (*n* = 9), anti-hPVRIG mAb (*n* = 9) or isotype-matched control mAb (mouse IgG) (*n* = 7) intraperitoneally (i.p.) every three days starting on day 8 for five times. And median tumor size was shown on the right. Each symbol represents an individual health donor (**d**, **e**, **g**) or B-NDG mouse (**i**, **j**). Data are representative of at least two independent experiments. Error bars represent means ± s.e.m. Statistical significance was determined using unpaired two-tailed *t* test (**b**, **c**, **f**, **g**), paired two-tailed *t* test (**e**) or two-way ANNOVA (**i**, **j**). *p < 0.05; **p < 0.01; ***p < 0.001; ****p < 0.0001

To analyze the expression of PVRIG on human NK cells, we evaluated the surface expression of PVRIG on NKG cell line, a highly cytotoxic human NK cell line established in our laboratory [32]. We found that almost all NKG cells expressed PVRIG (Fig. 7a). To better investigate the role of PVRIG on human NK cells, we generated mouse anti-human PVRIG mAb (Clone 2) that effectively blocks the interaction between human PVRIG and its ligand PVRL2 (Fig. S7a–e). Anti-hPVRIG mAb significantly enhanced the expression of effector molecules (NKG2D, CD107a and perforin) of NKG cells in co-culture with SW620 colon cancer cells (Fig. 7b) and improved their cytotoxicity against SW620 cells at effector/target ratios of 1:1 and 5:1 in vitro (Fig. 7c). Consistent with previous report [24], we found that human primary NK cells expressed much higher PVRIG (72.13% ± 1.348%) than CD8^+^ T cells (26.44% ± 1.023%) by analyzing PVRIG expression on human PBMCs (Fig. 7d). Anti-hPVRIG mAb significantly enhanced the cytokine secretion (IFN-γ and TNF-α) and degranulation (CD107a) of NK cells from PBMCs in co-culture with SW620 cells (Fig. 7e) and significantly improved the cytotoxicity of purified NK cells against SW620 tumor cells at indicated effector/target ratios in vitro (Fig. 7f). In addition, enhanced lysis of SW620 colon cancer cells, A375 melanoma cells or SK-OV-3 ovarian cancer cells by human PBMCs in vitro was observed upon treatment with anti-hPVRIG mAb (Fig. 7g).

To evaluate the therapeutic effect of anti-hPVRIG mAb in vivo, we inoculated B-NDG mice (which lack T, B and NK cells) with SW620 colon cancer cells subcutaneously on day 0. Mice were grouped randomly and received expanded NK cell transfer on days 7, 12 and 17, along with anti-hPVRIG mAb or mIgG treatment on days 7, 10, 13, 16 and 19. These expanded human NK cells expressed high level of PVRIG (Fig. 7h). Consistent with our results in *Rag1*^*−/−*^ mice, we observed significantly slower tumor growth (*P* < 0.05) in humanized tumor-bearing mice treated with anti-hPVRIG mAb compared to those treated with control antibody (Fig. 7i), indicating that PVRIG blockade in humanized mice could benefit directly via NK cells. Furthermore, we also reconstituted xenograft models using human PBMCs. Consistent with our results in NK cell-reconstituted xenograft models, we observed significantly slower tumor growth (*P* < 0.001) in humanized tumor-bearing mice treated with anti-hPVRIG mAb (Fig. 7j). Taken together, our results demonstrated that anti-hPVRIG mAb in vitro enhanced the cytotoxicity of human NKG cells, purified NK cells and PBMCs against human tumor cells and, further, significantly inhibited tumor growth in human NK cell- or PBMC-reconstituted xenograft mice*,* suggesting the potential value of PVRIG blockade in translational applications.

## Discussion

Chemotherapies and radiotherapies are effective against majority of tumors; however, they also bring many unpleasant side effects. Unlike traditional anti-tumor therapies, immunotherapies involving checkpoint blockade have gained their credit due to specific targeting and less side effects and have revolutionized the ways to treat cancer [1]. Although a variety of checkpoint inhibitors targeting CLTA-4, PD-1 and PD-L1 have shown impressive potent anti-tumor immunity and durable responses in some patients, there is still a large percentage of patients who do not respond to and therefore cannot benefit from these treatments [37], emphasizing the importance of finding novel immune checkpoint targets or combinational strategies.

Nectin and nectin-like families are important cell–cell adhesion molecules belonging to the immunoglobulin superfamily [38]. Some nectin family molecules can interact with receptors on the surface of immune cells to participate in immune regulation [11]. The co-inhibitory receptors CD96, TIGIT, PVRIG and the costimulatory receptor CD226 comprise a critical regulatory system for lymphocyte activity and anti-tumor immunity, and they share the same ligands CD155 and PVRL2[14]. Engagement of CD226 with its ligands PVRL2 and CD155 on target cells is essential for enhancing the anti-viral and anti-tumor functions of NK cells and T cells [39, 40], whereas CD96, TIGIT and PVRIG counterbalance CD226-dependent lymphocyte activation. Blockade of CD96 reduces the experimental and spontaneous metastases dependent of CD226 and INF-γ [21, 41]. Anti-TIGIT antibody mediates the rescue of anti-tumor responses of effector T cells that requires the costimulatory signal of CD226 [16, 42]. TIGIT preferentially binds to CD155, whereas PVRIG is the main inhibitory receptor for PVRL2 [24, 43]. PVRIG binds to PVRL2 with a higher affinity than CD226 [24], and blockade of PVRIG/PVRL2 interaction is very likely to result in the dimmed inhibitory effect of PVRIG and enhanced activating effect of CD226.

The latest research has shown that PVRIG is highly expressed on the terminally exhausted CD8^+^ T cells, suggesting that PVRIG is important in the exhaustion of CD8^+^ T cells [25]. Indeed, blockade of PVRIG restores the proliferation and cytokine production of T cells [24, 36], and genetic deletion of PVRIG enhances IFN-γ production of tumor-infiltrating CD8^+^ T cell and slows the tumor growth [26]. Consistent with their study, we showed that both genetic deficiency and blockade of PVRIG could enhance the cytotoxicity and IFN-γ production of tumor-infiltrating CD8^+^ T cells in MC38 tumor-bearing mice.

Besides CD8^+^ T cells, NK cells are also essential in anti-tumor immunity. The role of NK cells should not be underestimated. Previous studies have reported that blocking PVRIG enhances the ADCC function of human NK cells [30] and cytotoxicity against PVRL2^hi^PVR^lo^ acute myeloid leukemia target cells [31]. However, the role of PVRIG on NK cells in tumor microenvironment has not been investigated. In our study, we used three in vivo murine tumor models to show that tumor-infiltrating PVRIG^+^ NK cells are more exhausted (higher CD96, TIGIT, Tim-3, PD-1 and NKG2A expression) compared with PVRIG^−^ tumor-infiltrating NK cells (with the exception of NKG2A on MCA205 tumor model-derived PVRIG^+^ NK cells). CD96, TIGIT, Tim-3, PD-1 and NKG2A are typical NK cell exhaustion-related markers [44], and their upregulation (both in percentage and MFI) indicated the potential exhaustion or exhausted phenotype of PVRIG^+^ NK cells. Genetic knock-out of PVRIG or pharmacologic blockade of PVRIG/PVRL2 interaction significantly inhibited the exhaustion of NK cells and enhanced their cytotoxicity and IFN-γ production and therefore limited the subcutaneous tumor growth and prolonged the survival of tumor-bearing mice. By depletion of NK cells or/and CD8^+^ T cells, respectively, we showed that NK cells and CD8^+^ T cells both contributed to the therapeutic effects of PVRIG blockade in MC38 tumor model. Concurrent depletion of NK cells and CD8^+^ T cells resulted in larger tumor size and shorter survival time in tumor-bearing mice treated with anti-PVRIG mAb. Furthermore, by constructing MC38 tumor model using *Rag1*^*−/−*^ mice (which lack T cells and B cells), we showed that anti-PVRIG mAb was effective even in the absence of adaptive immunity, suggesting the importance of NK cells in PVRIG-targeted treatments.

It was reported previously that treatment with anti-PVRIG mAb alone shows no inhibitory effect on tumor growth in murine CT26 colon cancer model [26], consistent with their study, we observed no influence of anti-PVRIG mAb on CT26 tumor-bearing mice (data not shown). However, both early (when tumor size is around 10 mm^3^) and late (when tumor size is around 100–150 mm^3^) treatments with our anti-PVRIG mAb alone were effective against MC38 tumors in vivo, resulting in significant inhibition of tumor growth and prolonged survival of tumor-bearing mice. The potential effectiveness of PVRIG blockade in CT26 colon cancer can be boosted to significantly reduce tumor burden by combined blockade with anti-PD-L1[26], suggesting that combinational therapies can be more effective [45]. Indeed, our study showed that combined blockade of PVRIG and PD-L1 significantly reduced tumor size in MC38 tumor-bearing mice and resulted in better therapeutic effects than using either mAb alone. Given the importance of CD226 in the lysis of tumor cells, combinational therapies based on the blockade of inhibitory receptors in this family could be very attractive. As reported, blocking CD96 in *Tigit*^*−/−*^ mice is more effective than in wild-type mice in experimental and spontaneous lung metastasis models [21]. Blockade of PVRIG with TIGIT effectively enhances the cytokine production and cytotoxicity of human CD8^+^ T cells in vitro [36]. In addition, combined blockade of PVRIG and TIGIT shows a better performance in promoting human NK cell ADCC function triggered by trastuzumab than blocking either one alone [30]. A recent study has also proved that combined blockade of PVRIG and TIGIT further improves the activation and cytotoxicity of NK cells [31]. These results indicate the potential of PVRIG in combined blockade, and triple combination involving PVRIG (with anti-TIGIT and anti-PD-1) will also launch soon.

COM701, a first-in-class therapeutic antibody targeting human PVRIG generated by Compugen, shows early signs of efficacy, both as a monotherapy and in combination with the PD-1 inhibitor nivolumab in patients with a variety of advanced solid tumors in a Phase I clinical trial [46]. Here, the mouse anti-human PVRIG mAb generated in our study could entirely block the interaction between PVRIG and PVRL2 with high binding affinity (4 pM) to human PVRIG. The blockade of PVRIG using this mAb enhanced the cytokine secretion and cytotoxicity of human NK cells against various tumor cell lines in vitro. Furthermore, we used human NK cell- or PBMC-reconstituted xenograft model to verify the anti-tumor efficacy of anti-hPVRIG in vivo. As expected, blocking human PVRIG significantly reduced the tumor size in NK cell-reconstituted xenograft mice, suggesting that PVRIG blockade could be effective by acting only on NK cells. The PBMC-reconstituted xenograft model showed similar results, in which the use of anti-hPVRIG significantly reduced tumor size in PBMC-reconstituted xenograft mice, again, proving the anti-tumor efficacy of anti-hPVRIG mAb generated in our laboratory.

## Conclusions

In summary, our study, for the first time to our knowledge, illustrated that tumor-infiltrating PVRIG^+^ NK cells were exhausted in tumor microenvironment. Further, by using PVRIG-deficient mice and self-generated anti-mouse PVRIG mAb, we showed that PVRIG deficiency or PVRIG blockade (both early and late treatments) could reduce the tumor size and prolong the survival of tumor-bearing mice through inhibiting NK cell and CD8^+^ T cell exhaustion. Our study also provided novel findings on the roles of NK cells during PVRIG blockade in vivo. We showed that NK cells and CD8^+^ T cells both contributed to the therapeutic effect of PVRIG blockade, since depletion of either one resulted in the impaired effect of anti-PVRIG mAb. By using *Rag1*^*−/−*^ mice, we further proved that PVRIG blockade could still be effective even in the absence of adaptive immunity. Furthermore, we used human NK cell- or PBMC-reconstituted xenograft model to show the effect of blocking human PVRIG. Indeed, blocking human PVRIG in tumor-bearing humanized mice resulted in smaller tumor sizes, indicating the therapeutic value of anti-hPVRIG mAb, meanwhile, pointing out that anti-hPVRIG mAb could be effective by acting only on NK cells. Collectively, our study suggests that targeting PVRIG with therapeutic mAb can be a promising way to treat cancer via reversing exhaustion of both NK cells and CTLs, from which we highlight the importance of NK cells in PVRIG blockade and suggest that more attention needs to be paid on NK cells in PVRIG-targeted treatments.

## Supplementary Information

**Additional file1**. Supplementary figures and tables.

## Data Availability

All data relevant to the study are included in the article or uploaded as supplementary information.

## Abbreviations

PVRIG: Poliovirus receptor-related immunoglobulin domain-containing protein
PVRL2: Poliovirus receptor-related protein 2
CD112R: CD112 receptor
NK cell: Natural killer cell
TIGIT: T cell immunoreceptor with Ig and ITIM domains
CTL: Cytotoxic T lymphocyte
ADCC: Antibody-dependent cell-mediated cytotoxicity
PBMC: Peripheral blood mononuclear cell
mAb: Monoclonal antibody
TIL: Tumor-infiltrating lymphocyte
COAD: Colon adenocarcinoma
