# Supplementary material for: Blockade of checkpoint receptor PVRIG unleashes anti-tumor immunity of NK cells in murine and human solid tumors
Source: J Hematol Oncol. 2021 Jun 26;14:100. doi: 10.1186/s13045-021-01112-3 (PMC8236157; doi:10.1186/s13045-021-01112-3)
Supplement: Supplementary file 1 — Additional file1. Supplementary figures and tables. [file 13045_2021_1112_MOESM1_ESM.docx]

**Additional file 1**


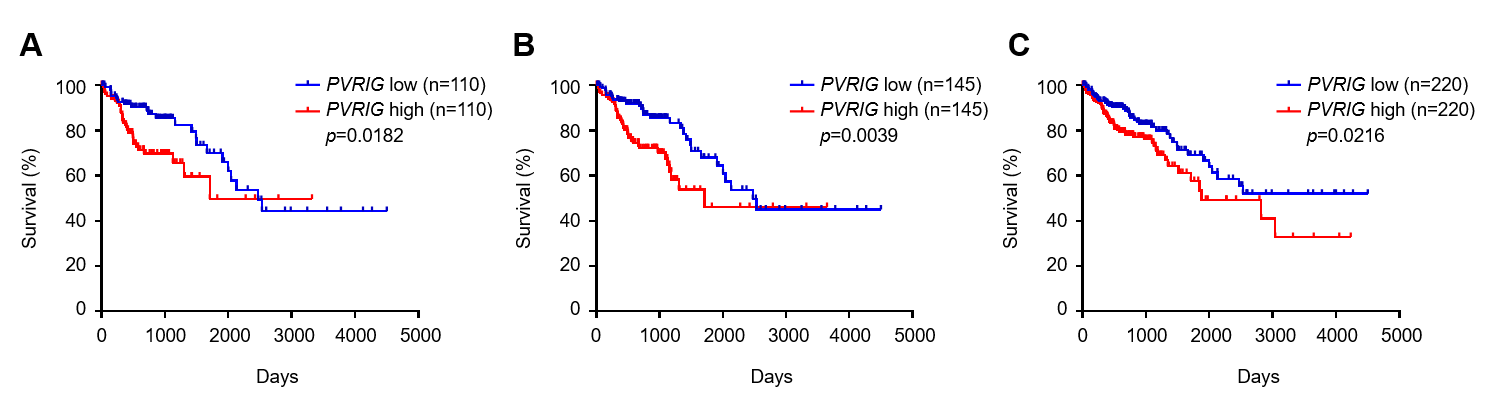


**Additional file 1: Figure S1. PVRIG is associated with poor clinical outcomes in patients with colon adenocarcinoma.**

Kaplan-Meier survival of COAD patients (A Cut off: 25-25; B Cut off: 33-33; C Cut off: 50-50) based on the level of PVRIG mRNA expression (PVRIG low, blue; PVRIG high, red). Data were analyzed for significance by the log-rank test.


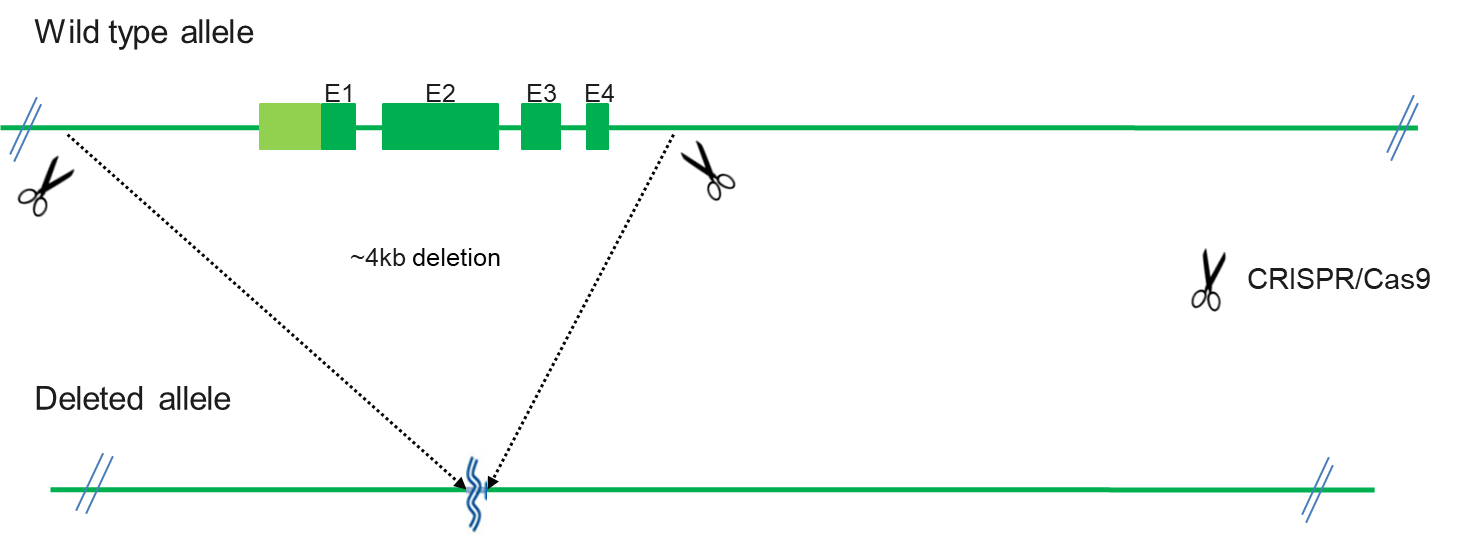


**Additional file 1: Figure S2. Generation of PVRIG-deficient mice.**

The PVRIG knockout mice were generated by Biocytogen Co. Ltd. (Beijing, China). Two sgRNAs were designed to delete the exons 1 to 4 of *Pvrig* gene locus in the mouse genome.


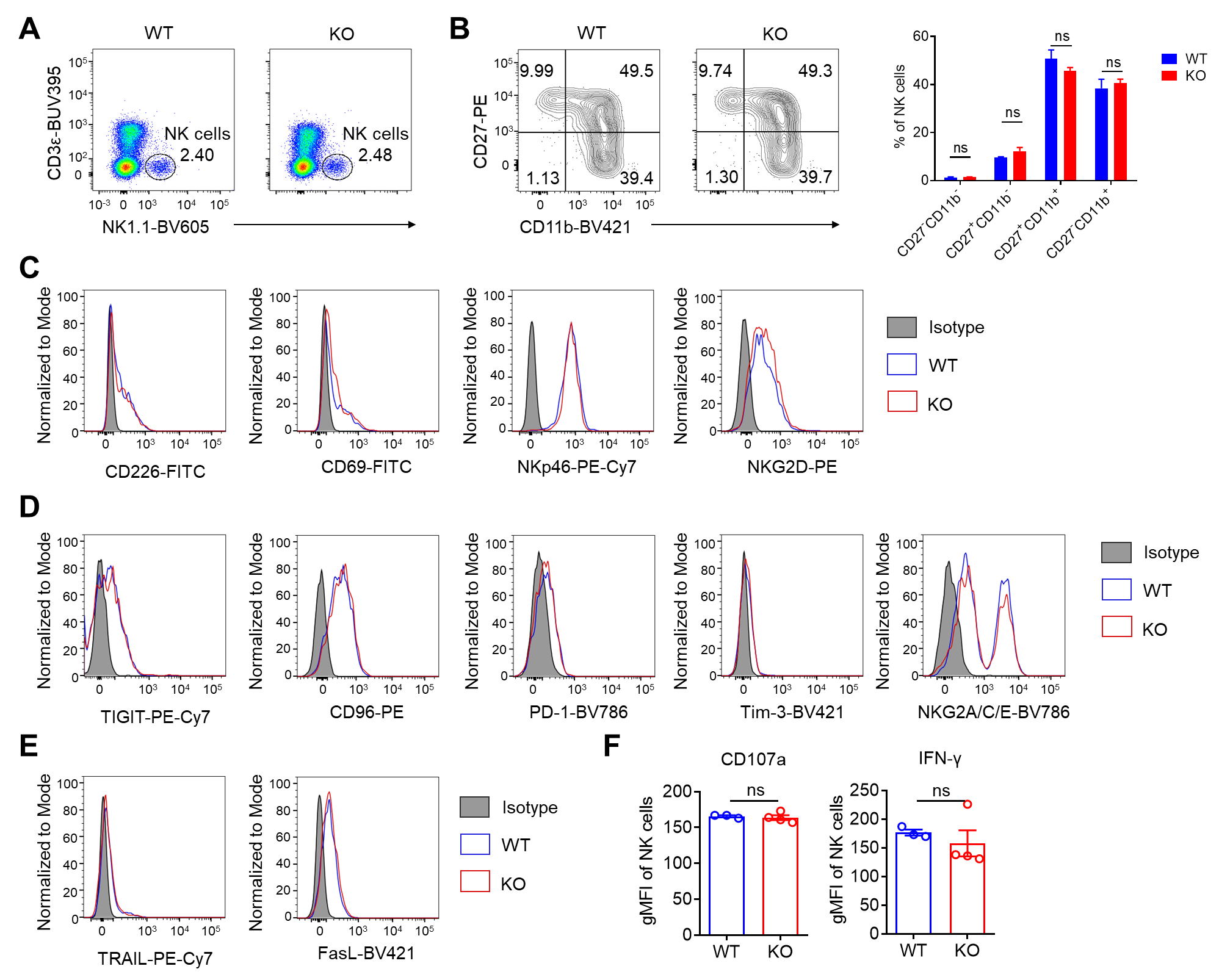


**Additional file 1: Figure S3. NK cells of PVRIG-deficient mice are phenotypically and functionally consistent with those of WT mice under steady state.**

(A) Representative FACS plot of CD3e^-^NK1.1^+^ NK cells in the spleen of WT and PVRIG KO mice. (B) Representative FACS plot of CD11b and CD27 expression in WT and PVRIG KO mice splenic NK cells. Quantitation of NK cells in different stages of maturation was shown on the right. (C) Representative histograms of CD226, CD69, NKp46 and NKG2D expression in WT and PVRIG KO mice splenic NK cells. (D) Representative histograms of TIGIT, CD96, PD-1, Tim-3 and NKG2A/C/E expression in WT and PVRIG KO mice splenic NK cells. (E) Representative histograms of TRAIL and FasL expression in WT and PVRIG KO mice splenic NK cells. (F) Geometric MFI of CD107a and IFN-γ of WT (n=3) and PVRIG KO (n=4) mice splenic NK cells. Each symbol represents an individual mouse (F). Data were representative of two independent experiments. Error bars represent means ± s.e.m. Statistical significance was determined using unpaired two-tailed *t* test (C, D). ns, not significant (p > 0.05).


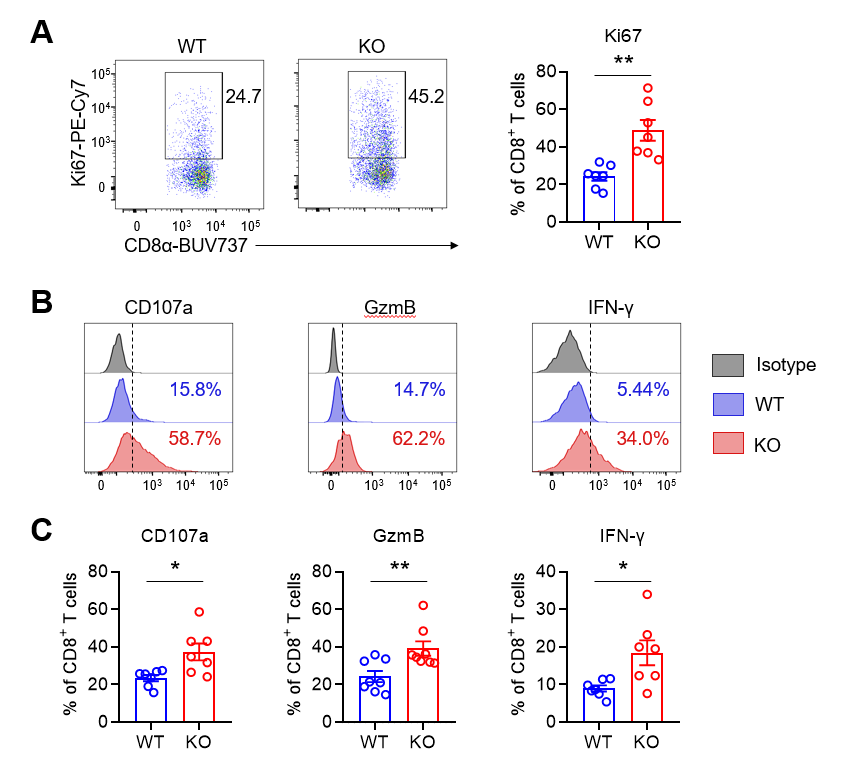


**Additional file 1: Figure S4. PVRIG deficiency prevents exhaustion of tumor-infiltrating CD8^+^ T cells in tumor-bearing mice.**

(A) Representative flow plots (left) and quantification (right) of Ki67^+^ tumor-infiltrating CD8^+^ T cells in WT and PVRIG KO mice (n=7 per group). (B, C) Representative histograms (B) and quantification (C) of CD107a, Granzyme B (GzmB) and IFN-γ expression of tumor-infiltrating CD8^+^ T cells in WT and PVRIG KO mice (n=7or 8 per group). Each symbol represents an individual mouse. Data were representative of at least two independent experiments. Error bars represent means ± s.e.m. Statistical significance was determined using unpaired two-tailed *t* test. *p < 0.05, **p < 0.01.


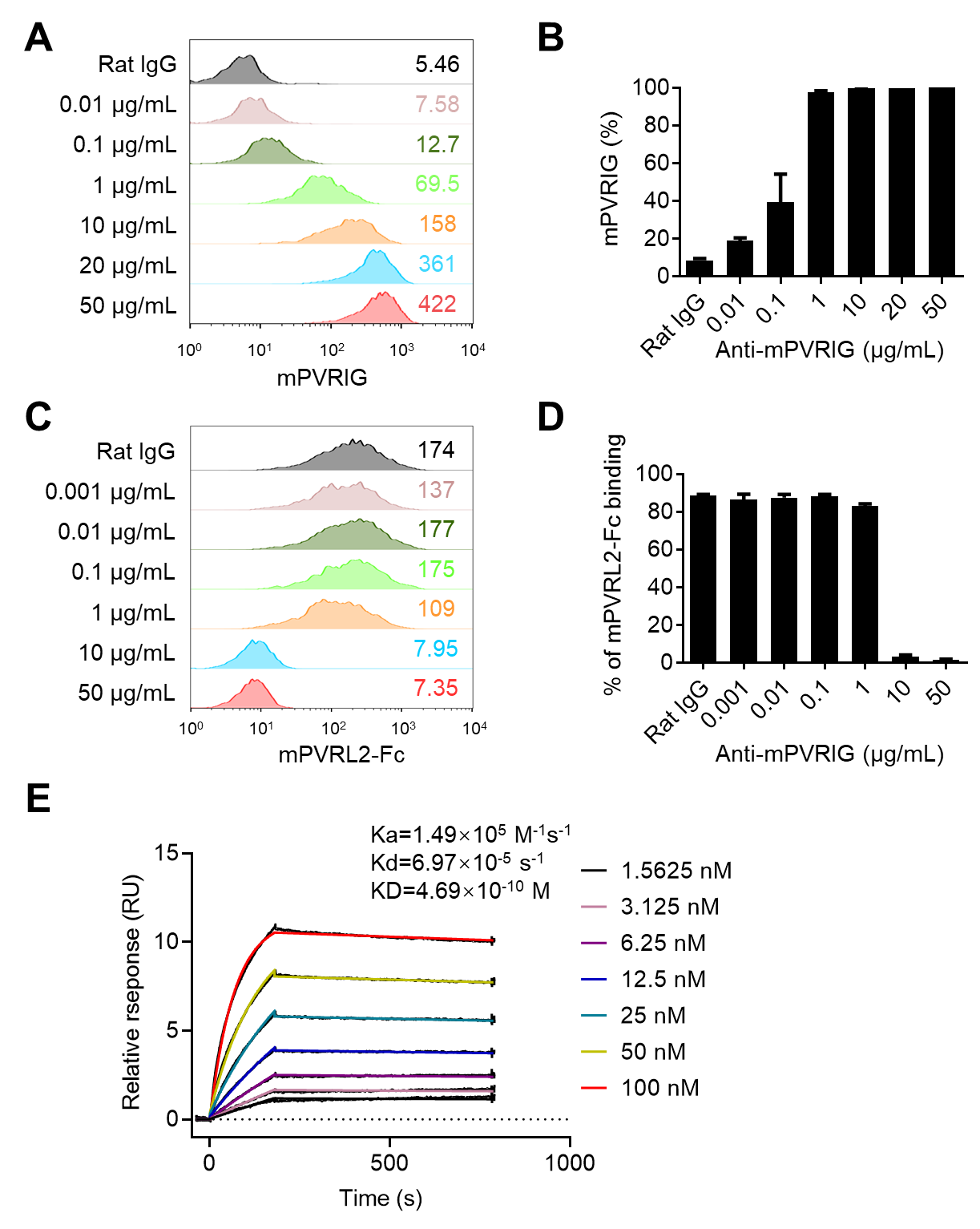


**Additional file 1: Figure S5. Identification of rat anti-mouse PVRIG monoclonal antibody.**

(A, B) Representative histogram (A) and quantification (B) of anti-mouse PVRIG mAb binding to mouse PVRIG-overexpressing 293T cells at indicated concentrations. Numbers indicate the geometric mean fluorescence intensity. (C, D) Representative histogram (C) and quantification (D) of mouse PVRL2-Fc fusion protein binding to mouse PVRIG-overexpressing 293T cells at indicated anti-mPVRIG concentrations. Numbers indicate the geometric mean fluorescence intensity. (E) Binding between anti-mouse PVRIG mAb and mouse PVRIG-Fc fusion protein was assessed by surface plasmon resonance (SPR). Data were representative of at least two independent experiments. Error bars represent means ± s.e.m.


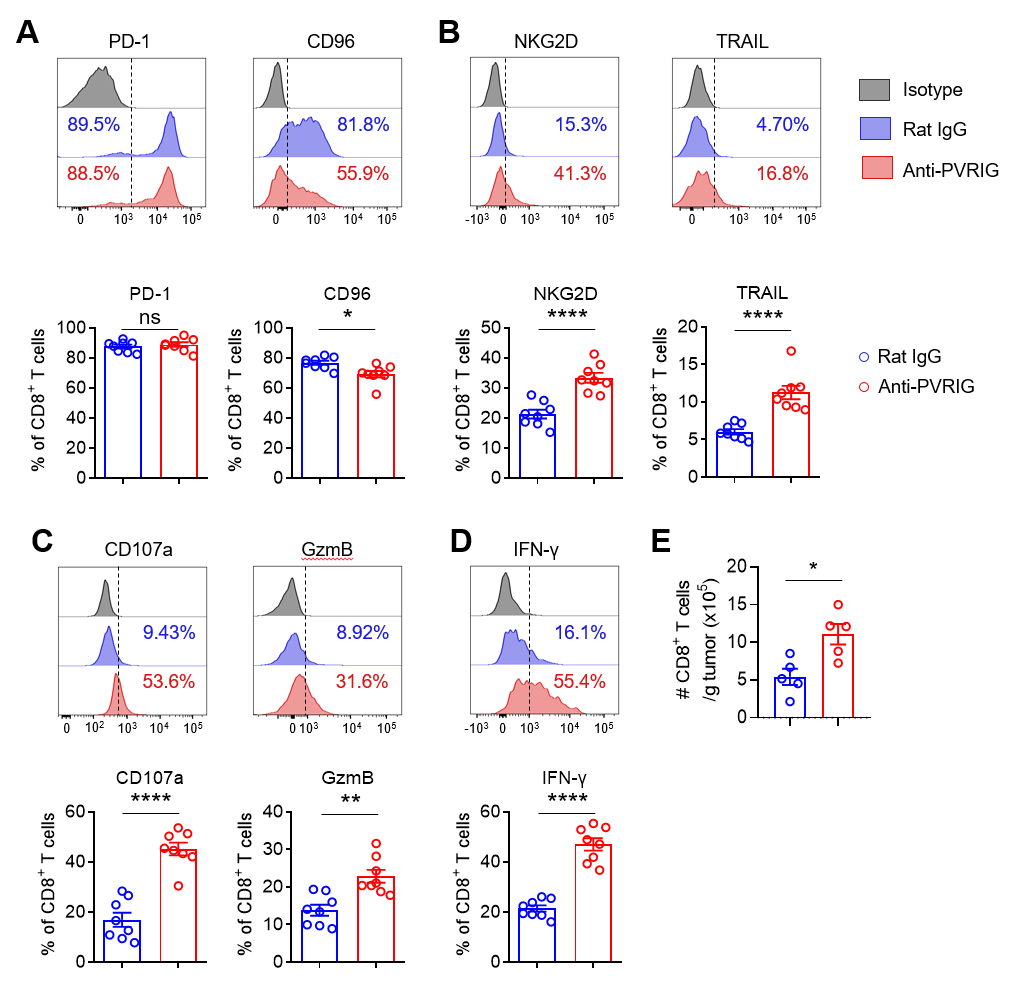


**Additional file 1: Figure S6. Blockade of PVRIG reverses exhaustion of tumor-infiltrating CD8^+^ T cells in tumor-bearing mice.**

Mice were injected with PBS, anti-PVRIG mAb or isotype-matched control mAb (rat IgG) intraperitoneally (i.p.) at various time points after injection of 5×10^4^ MC38 tumor cells subcutaneously (s.c.) on day 0 and sacrificed on day 28 after challenge as describe in Figure 4A. (A) Representative histograms (top) and quantification (bottom) of PD-1 and CD96 expression in tumor-infiltrating CD8^+^ T cells. (B) Representative histograms (top) and quantification (bottom) of NKG2D and TRAIL expression in tumor-infiltrating CD8^+^ T cells. (C) Representative histograms (top) and quantification (bottom) of CD107a and Granzyme B (GzmB) expression in tumor-infiltrating CD8^+^ T cells. (D) Representative histograms (top) and quantification (bottom) of IFN-γ synthesis in tumor-infiltrating CD8^+^ T cells. (E) Absolute number of tumor-infiltrating CD8^+^ T cells in mice treated with rat IgG or anti-PVRIG mAb. Each symbol represents an individual mouse (n=8 (A-D) or n=5 (E) per group). Data were representative of two independent experiments. Error bars represent means ± s.e.m. Statistical significance was determined using unpaired two-tailed *t* test (A-E). ns, p>0.05, *p < 0.05, **p < 0.01, ***p < 0.001 and ****p < 0.0001.


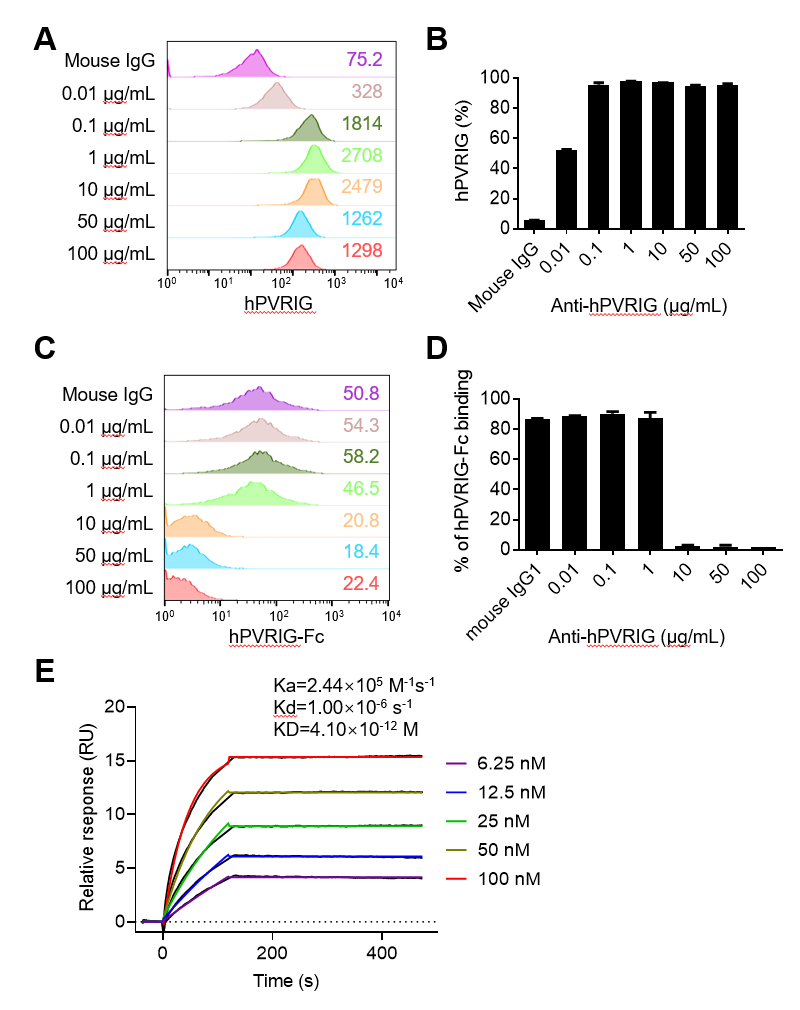


**Additional file 1: Figure S7. Identification of mouse anti-human PVRIG monoclonal antibody.**

(A, B) Representative histogram (A) and quantification (B) of anti-human PVRIG mAb binding to human PVRIG-overexpressing 293T cells at indicated concentrations. Numbers indicate the geometric mean fluorescence intensity. (C, D) Representative histogram (C) and quantification (D) of human PVRIG-Fc fusion protein binding to human PVRL2-overexpressing 293T cells at indicated anti-hPVRIG concentrations. Numbers indicate the geometric mean fluorescence intensity. (E) Binding between anti-human PVRIG mAb and human PVRIG-Fc fusion protein was assessed by surface plasmon resonance (SPR). Data were representative of two independent experiments. Error bars represent means ± s.e.m.

**Additional file 1: Table S1**. **Details of patients with colon adenocarcinoma (COAD).**

| Patient ID | Sample | Age | Gender | Stage | Prior treatment |
| --- | --- | --- | --- | --- | --- |
| COAD-1 | Primary tumor | 78 | Male | Ⅳ | Untreated |
| COAD-2 | Primary tumor | 83 | Female | Ⅰ | Untreated |
| COAD-3 | Primary tumor | 77 | Female | Ⅰ | Untreated |
| COAD-4 | Primary tumor | 57 | Male | Ⅱ | Untreated |
| COAD-5 | Primary tumor | 78 | Female | Ⅱ | Untreated |
| COAD-6 | Primary tumor | 66 | Male | Ⅰ | Untreated |
